# Transgelin Promotes Glioblastoma Stem Cell Hypoxic Responses and Maintenance Through p53 Acetylation

**DOI:** 10.1002/advs.202305620

**Published:** 2023-12-12

**Authors:** Huan Li, Chao Song, Yang Zhang, Guohao Liu, Hailong Mi, Yachao Li, Zhiye Chen, Xiaoyu Ma, Po Zhang, Lidong Cheng, Peng Peng, Hongtao Zhu, Zirong Chen, Minhai Dong, Sui Chen, Hao Meng, QunGen Xiao, Honglian Li, Qiulian Wu, Baofeng Wang, Suojun Zhang, Kai Shu, Feng Wan, Dongsheng Guo, Wenchao Zhou, Lin Zhou, Feng Mao, Jeremy N. Rich, Xingjiang Yu

**Affiliations:** ^1^ Department of Histology and Embryology School of Basic Medicine Tongji Medical College Huazhong University of Science and Technology Wuhan 430030 China; ^2^ Department of Neurosurgery Tongji Hospital Tongji Medical College Huazhong University of Science and Technology Wuhan 430030 China; ^3^ Intelligent Pathology Institute The First Affiliated Hospital of USTC Division of Life Sciences and Medicine University of Science and Technology of China Hefei 230031 China; ^4^ UPMC Hillman Cancer Center Department of Medicine University of Pittsburgh Medical Center Pittsburgh PA 15219 USA; ^5^ Department of Neurology University of Pittsburgh School of Medicine Pittsburgh PA 15213 USA

**Keywords:** glioblastoma stem cells, HDAC2, HIF1α Hypoxia, natural borneol, sodium valproate, transgelin

## Abstract

Glioblastoma (GBM) is a lethal cancer characterized by hypervascularity and necrosis associated with hypoxia. Here, it is found that hypoxia preferentially induces the actin‐binding protein, Transgelin (TAGLN), in GBM stem cells (GSCs). Mechanistically, TAGLN regulates HIF1α transcription and stabilizes HDAC2 to deacetylate p53 and maintain GSC self‐renewal. To translate these findings into preclinical therapeutic paradigm, it is found that sodium valproate (VPA) is a specific inhibitor of TAGLN/HDAC2 function, with augmented efficacy when combined with natural borneol (NB) in vivo. Thus, TAGLN promotes cancer stem cell survival in hypoxia and informs a novel therapeutic paradigm.

## Graphical abstract

Here, it is found that TAGLN, which is preferentially expressed in GSC, supports the hypoxic response of GSC by binding to HIF1α to form a complex. Mechanistically, TAGLN stabilizes HDAC2 to deacetylate p53 and maintains GSC self‐renewal under hypoxia. Inhibition of TAGLN/HDAC2‐p53 cell cycle axis by sodium valproate combined with natural borneol prolongs the survival of GBM experimental animal models.

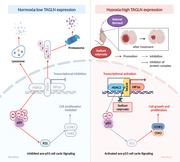

## 1 Introduction

Glioblastoma (GBM) is the most prevalent primary intrinsic malignant brain tumor with conventional therapies offering only palliation.^[^ ^1^ ^]^ GBM was one of the first cancers to be comprehensively genetically characterized with the p53 pathway among the core molecular pathways.^[^ ^2^ ^]^ p53 is a canonical tumor suppressor and transcription factor frequently mutated in tumor cells exposed to hypoxic microenvironments and regulates cell cycle progression and survival.^[^ ^3^ ^]^ Dysfunction of p53 and cell cycle regulators contributes to tumor cell proliferation, migration, and stemness.^[^ ^4^ ^]^ Post‐translational modifications (PTMs) of p53 include ubiquitination, phosphorylation, and acetylation; PTMs direct p53 protein stabilization and activation. Acetylation is essential for basic p53 function, and multiple sites of p53 (including lysines K370, K372, K373, K381, K382, and K386) are acetylated or deacetylated to define the overall level of p53 acetylation.^[^ ^5^ ^]^ p53 acetylation is crucial for its tumor suppressive functions, and deacetylase inhibitors have shown preclinical efficacy in multiple cancer types, including GBM.^[^ ^6^ ^]^

The prior designation of GBM as glioblastoma multiforme belies its striking intratumoral diversity. Neoplastic cells within GBMs display hierarchies of cellular differentiation with stem‐like, cancer stem cells at the apex. GBM stem cells (GSCs) contribute to tumor initiation, maintenance, and therapeutic resistance.^[^ ^7^ ^]^ GBMs are defined by morphopathological features, including pseudopallisading necrosis, hypoxic and inflammatory regions bordered by tumor cells, including GSCs, which contribute to tumor recurrence and invasion into normal brain.^[^ ^8^ ^]^ Hypoxia and hypoxia‐inducible factors (HIFs) induce cancer stem cell markers and maintain GSC self‐renewal and proliferation.^[^ ^9^ ^]^ The HIF1α/STAT3 coactivation complex induces VASORIN to stabilize NOTCH1 and promote GSC growth.^[^ ^10^ ^]^ Based on the negative prognosis associated with hypoxia and its role in promoting GSC growth, we hypothesized that identification of novel hypoxia‐inducible molecular targets in GSCs may inform the development of novel treatments for this lethal cancer.

TAGLN, also called SM22, is a focal actin‐binding protein and a key protein in smooth muscle cells. Like transforming growth factor‐β (TGF‐β), which induces TAGLN^[^ ^11^ ^]^, TAGLN serves context‐specific roles in cancer with both tumor‐suppressive and oncogenic functions in select cancer types. TAGLN is expressed at low levels in bladder carcinoma^[^ ^12^ ^]^ and prostate cancer^[^ ^13^ ^]^, but potentiates the proliferation and growth of other cancer cells, which suggests that TAGLN might represent an early event in tumor progression. TAGLN is expressed at high levels in colorectal cancer^[^ ^14^ ^]^ and ovarian cancer^[^ ^15^ ^]^, contributing to tumor infiltration and migration at advanced tumor stages. In bladder cancer cells, TAGLN is regulated by p53 and PTEN (phosphatase and tensin homolog) to inhibit proliferation.^[^ ^12^ ^]^

Here, we show that GSCs express high levels of TAGLN relative to the other tumor cells, and TAGLN informs poor prognosis for GBM patients. Therefore, we investigated the contribution of TAGLN in GSCs adaptation to hypoxia and its downstream molecular functions.

## 2 Results

### 2.1 Gliomas Express Increased TAGLN

**Figure 1:**
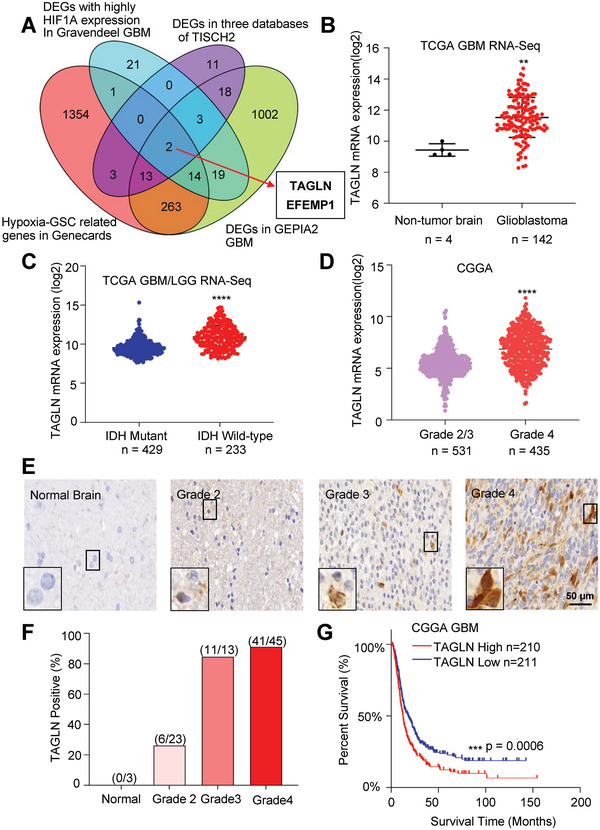
TAGLN is highly expressed in glioblastoma. A) Venn diagram showing the overlapping of four datasets, hypoxia and GSC‐related genes in Genecards, DEGs associated with high *HIF1A* expression,intersection of DEGs in three databases of TISCH2 and DGEs from GEPIA2 GBM, comprised two genes: *TAGLN* and *EFEMP1*. B) *TAGLN* expression in a panel of GBM (IDH‐WT) and normal brain samples from the Gliovis TCGA RNA‐seq dataset. Unpaired t‐tests were used to calculate statistical significance, *p*<0.01. C) Histogram of *TAGLN* expression in IDH wild‐type and mutant GBM samples from the Gliovis TCGA_GBM/LGG (IDH‐WT) RNA‐seq dataset. Unpaired t‐tests were used to calculate statistical significance, *p*<0.0001. D) Plot of *TAGLN* expression in patients from the CGGA (IDH‐WT) database (Student's t‐test). E) Representative images of glioma tissue microarray samples showing TAGLN expression. Scale bar, 50 µm. F) The proportion of TAGLN‐positive tissues in glioma or normal brain tissue was shown. G) Kaplan‐Meier survival analysis of patients with different *TAGLN* expression levels in the Gliovis CGGA (IDH‐WT) dataset (log‐rank test). Data are represented as mean ± SD (**p < 0.01, ***p < 0.001, and ****p < 0.0001).

To identify critical hypoxia‐related molecular targets in GBM, we prioritized genes that were highly expressed in GBMs relative to normal brains, GSCs within tumors, and the hypoxic microenvironment. Therefore, we leveraged three single‐cell RNA‐seq databases for each element from GEO for gliomas (GSE103224, GSE139448, and GSE141383) with TISCH2 (http://tisch.comp‐genomics.org/home/) and the overlapping differential genes (DEGs, *n =* 50, log_2_FC ≥ 1) (Figure S1A, Supporting Information). Next, we generated a Venn diagram illustrating the intersection between: 1) 50 associated genes in the malignant cells of GBM from TISCH2; 2) DEGs (*n =* 1334, log_2_FC >2) from GEPIA GBM (http://gepia.cancer‐pku.cn/); 3) DEGs (*n =* 1650, ≥ median) associated with hypoxia and GSCs in Genecards (https://www.genecards.org/); and 4) DEGs (*n =* 60, log_2_FC ≥ 1 and *p* ≤ 0.05) associated with *HIF1A* high expression in GlioVis Gravendeel_GBM. The intersection of the four datasets revealed two genes: *TAGLN* and *EFEMP1* (**Figure** **1A**). Both genes have been reported to promote the migration and infiltration of cancer cells, with EFEMP1 shown to promote GBM invasion.^[^ ^16^ ^]^ To interrogate TAGLN expression in multiple tumors, we mapped *TAGLN* expression across The Cancer Genome Atlas (TCGA) databases for multiple tumor types. Compared to normal tissues, most tumors expressed relatively lower expression of *TAGLN*, whereas GBM patient samples showed increased expression (Figure 1B; Figure S1B, Supporting Information). Mutations in isocitrate dehydrogenase (IDH) define a glioma subset with a favorable prognosis.^[^ ^4^, ^17^ ^]^ Gliomas with wild‐type IDH (IDH‐WT) expressed higher *TAGLN* levels than tumors harboring IDH mutations in the TCGA‐GBM/LGG (low‐grade glioma) RNA‐seq database (Figure 1C). Analysis of *TAGLN* expression in an independent glioma database, the Chinese Glioma Genome Atlas (CGGA), revealed that GBMs showed higher *TAGLN* expression than low‐grade gliomas (LGG), supporting an association with tumor grade (Figure 1D). To validate these findings at the protein level, we performed immunohistochemistry (IHC) for TAGLN on tissue arrays with glioma surgical specimens of various grades (grade 2 (*n =* 23), grade 3 (*n =* 13), and GBM (*n =* 45)) and normal brain tissues (*n =* 3). TAGLN was strongly expressed in glioma cells in 41 of 45 (91.1%) of GBMs, 11 of 13 (84.6%) of grade 3 gliomas, but in fewer than 30% of grade 2 gliomas, and was absent in normal brain tissues (Figure 1E,F). Consistently, immunoblotting (IB) validated high TAGLN expression in GBM patient samples (Figure S1C, Supporting Information). *TAGLN* expression correlated with poor patient prognosis in the CGGA GBM (IDH‐WT) and the TCGA GBM/LGG (IDH‐WT) databases (Figure 1G; Figure S1D, Supporting Information). Collectively, these data support TAGLN as a potential oncogenic factor in GBM.

### 2.2 Hypoxia Induces TAGLN in GSCs

**Figure 2:**
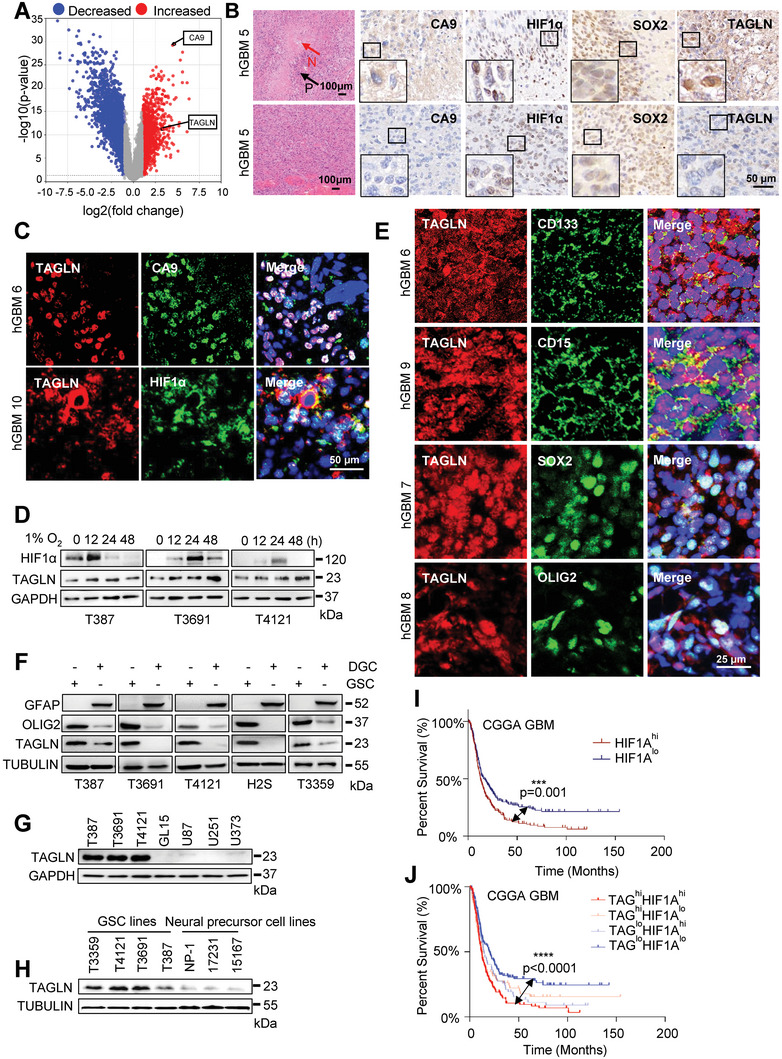
Preferential expression of TAGLN in hypoxia‐induced GSCs. A) Volcano plot of differentially expressed genes at peritumoral regions and pseudopalisades in GBM tissue. Red dots, genes upregulated in hypoxia; Blue dots, genes downregulated in hypoxia. B) Representative images of HE (Scale bar, 100 µm) and IHC (Scale bar, 50 µm) stained in pseudopalisades located around the necrosis (upper) and other areas (lower) of serial sections of hGBM samples. The red arrow points to the necrosis (N) in GBM and the black arrow represents the pseudopalisades (P) around the necrosis. The large black square in the lower‐left corner of the image is an enlarged view of the small black square in the image. C) Representative images of IF staining for TAGLN, CA9, and HIF1α in hGBM tissues. Scale bar, 50 µm. D) Expression levels of TAGLN and HIF1α were examined by IB in GSCs cultured in 1% O_2_ for the indicated time. E) Representative images of IF staining for TAGLN and putative GSCs markers in hGBM sections. Scale bar, 25µm. F) Cell lysates were analyzed by IB for TAGLN, OLIG2, and GFAP expression. G) The expression of TAGLN in GSCs and glioma cells was tested by IB. H) IB analyzed TAGLN expression in GSCs and NPCs. I) Kaplan‐Meier curve showing patient survival based on *HIF1A* mRNA expression in the CGGA GBM (IDH‐WT) dataset. J) The Kaplan‐Meier survival curve was calculated to measure survival in the group of *TAGLN* ^hi^ *HIF1A* ^hi^ (*n =* 139), *TAGLN* ^hi^ *HIF1A* ^lo^, (*n =* 73), *TAGLN* ^lo^ *HIF1A* ^hi^, (*n =* 73), and *TAGLN* ^lo^ *HIF1A* ^lo^ (*n =* 139).

Next, we investigated hypoxia regulation of TAGLN expression. The Ivy Glioblastoma Atlas Project (Ivy GAP) mapped regional gene expression in GBM tissues. *TAGLN* was highly expressed in regions of pseudopalisading necrosis compared to peritumoral regions of GBM (**Figure** **2A**). *TAGLN* expression positively correlated with hypoxia‐related genes (*HIF1A*, *CA9*, *LDHA*, *PDK1*, *PGK1*, *VEGFA*, and *EPAS1*) in the TCGA GBM/LGG (IDH‐WT) and CGGA GBM (IDH‐WT) datasets (Figure S2A,B, Supporting Information). IHC staining confirmed increased levels of hypoxic proteins (HIF1α and CA9) in pseudopalisades of GBM specimens, consistent with previous reports.^[^ ^9^, ^18^ ^]^ TAGLN was lowly expressed in other regions of GBM, while highly expressed in the pseudopalisades of GBM (Figure 2B; Figure S2C, Supporting Information). Co‐immunofluorescence (IF) staining of TAGLN, CA9, and HIF1α revealed that hypoxic regions of GBM samples preferentially expressed TAGLN (Figure 2C; Figure S2D, Supporting Information). To directly test the effects of hypoxia on TAGLN expression, we exposed a panel of patient‐derived GSCs to room air (21% O_2_) or hypoxia (1% O_2_) over a time course. Over 48 h, TAGLN expression increased in GSCs under hypoxia compared with normoxia (Figure 2D; Figure S2E, Supporting Information), suggesting that TAGLN is induced by hypoxia.

To determine the specificity of TAGLN expression in GSCs, we interrogated *TAGLN* expression in the Gliovis data portal to reveal a positive correlation between *TAGLN* and GSC markers (Figure S2F, Supporting Information). TAGLN co‐distributed with GSC markers CD133, CD15, SOX2, and OLIG2 in glioma cells (Figure 2E; Figure S2G, Supporting Information), suggesting that TAGLN was preferentially expressed in GSCs in patient tissues. To directly test this, we generated differentiated GBM cells (DGCs) from patient‐derived GSCs cultured in 10% serum for 14 days to induce differentiation. Serum induced the astrocyte marker, glial fibrillary acidic protein (GFAP), and reduced the expression of GSC markers, OLIG2 and SOX2. Using these matched cells, TAGLN was preferentially expressed in GSCs but not in matched DGCs (Figure 2F; Figure S2H, Supporting Information), bulk glioma cells (Figure 2G), or neural precursor cells (NPCs) (Figure 2H) under hypoxia. To further parse the impact of TAGLN and hypoxia, we segregated patients with GBMs based on levels of both *TAGLN* and *HIF1A* (high vs. low relative to the median) in GlioVis‐GBM (IDH‐WT). The median survival times (MST) of GBM patients with high *HIF1A* expression was 12.7 months, while that of patients with low *HIF1A* expression was 15.6 months, showing that GBM patients with high *HIF1A* expression had a poor prognosis, *p* = 0.001 (Figure 2I). Patients whose tumors had high expression levels of both *TAGLN* and *HIF1A* (MST = 12.2 months) had worse survival than those with low expression levels of both genes (MST = 20 months), *p*<0.0001 (Figure 2J), indicating that the effect is not only linked to *HIF1A* but also to *TAGLN*.

### 2.3 TAGLN Combines with HIF1α to Form a Hypoxic Transcriptional Activator Complex

**Figure 3:**
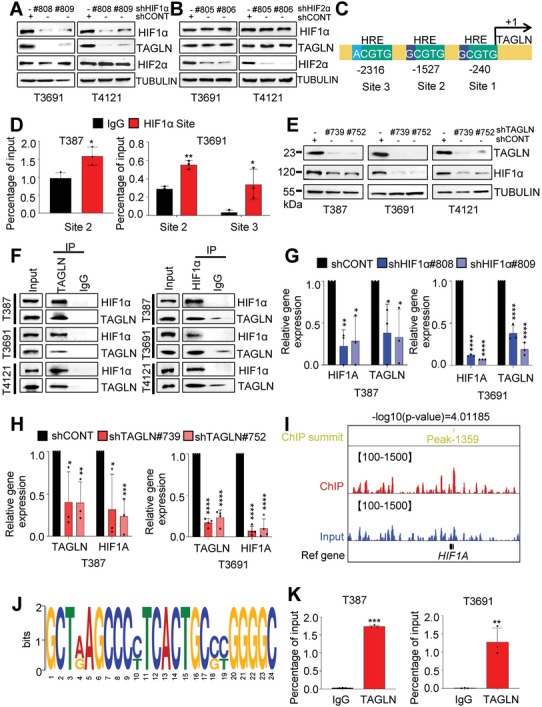
The hypoxic transcription factor‐TAGLN. A,B) IB showed the indicated protein levels in GSCs after targeting HIF1α or HIF2α. C) Schematic representation of the three predicted HRE‐binding sites in the *TAGLN* promoter. D) ChIP‐qPCR analysis of HRE site occupancy in *TAGLN* promoter (Student's t‐test). E) IB showed protein expression in GSCs after TAGLN targeting. F) Co‐IP analysis of endogenous TAGLN (left) or HIF1α (right) in GSCs, followed by determination of the expression of HIF1α and TAGLN by IB. G) mRNA expression of *HIF1A* and *TAGLN* was assessed by qRT‐PCR following *HIF1A* knockdown. H) Expression of *TAGLN* and *HIF1A* after targeting *TAGLN* in GSCs was examined by qRT‐PCR. I) TAGLN‐binding peak in *HIF1A*. J) *HIF1A* motif to which TAGLN binds. K) ChIP‐qPCR of the indicated genes in GSCs (Student's t‐test). Values in (D,G,H, and K) represent the mean ± SD from three independent experiments (*p < 0.05, **p < 0.01, ***p < 0.001, and ****p < 0.0001).

Given the central role of the HIF transcription factors in cellular responses to hypoxia, patient‐derived GSCs were transduced with lentiviruses encoding shRNAs with either a non‐targeting control sequence or targeting *HIF1A* or *HIF2A* (*EPAS1*) then cultured under hypoxia for 48 h. Upon HIF1α silencing, TAGLN was no longer induced by hypoxia (**Figure** **3A**), whereas targeting HIF2α did not alter TAGLN expression (Figure 3B). HIFs selectively bind to the core binding motif 5’‐A/GCGTG‐3’ located in gene promoters or intragenic regions to transcriptionally activate the expression of hypoxia‐inducible genes.^[^ ^19^ ^]^ To confirm the role of *HIF1A* in TAGLN regulation, we analyzed the *TAGLN* promoter region, which covered a ≈3 kb tiled array surrounding the transcription start site, and predicted three possible hypoxic response element‐binding sites (designated as binding sites 1, 2, and 3) by checking the UCSC Genome Browser Home (Figure 3C). Chromatin immunoprecipitation coupled with qRT‐PCR (ChIP‐PCR) identified HIF1α binding to site 2 in T387 GSCs and sites 2 and 3 in T3691 GSCs in the *TAGLN* promoter and induction of *TAGLN* expression under hypoxia (Figure 3D). Next, we explored the effect on HIF1α expression in GSCs following TAGLN knockdown or overexpression. Immunoblotting indicated that the expression of HIF1α was consistent with that of TAGLN (Figure 3E; Figure S3A, Supporting Information). In GBM patient specimens, TAGLN co‐localized with HIF1α on IF (Figure 2C; Figure S2D, Supporting Information).

TAGLN has been detected in the nucleus of colon cancer cells, where it interacts with transcription factors and also participates extensively in the transcription of genes.^[^ ^20^ ^]^ Therefore, we hypothesized that TAGLN may directly bind HIF1α. We performed reciprocal co‐immunoprecipitation (Co‐IP) experiments in GSCs, revealing binding between TAGLN and HIF1α (Figure 3F). Targeting *HIF1A*, but not *HIF2A* (*EPAS1*), reduced TAGLN transcript levels (Figure 3G; Figure S3B, Supporting Information). Reciprocally, *TAGLN* regulated *HIF1A* transcription with a >70% reduction in *HIF1A* transcript levels after TAGLN targeting (Figure 3H). In gain‐of‐function studies, TAGLN overexpression induced elevated *HIF1A* mRNA levels (Figure S3C, Supporting Information). ChIP‐seq showed that TAGLN is bound to the *HIF1A* promoter region in GSCs (Figure 3I). Multiple Em for motif elicitation analysis showed enrichment of elements within TAGLN‐binding sites (Figure 3J). We confirmed the presence of a unique TAGLN binding site in the upstream promoter of *HIF1A* (Figure 3K). Collectively, TAGLN forms a regulatory feedforward circuit with HIF1A to support the hypoxic responses of GSCs.

### 2.4 TAGLN Maintains GSC Self‐Renewal and Tumor Initiation With Hypoxia

**Figure 4:**
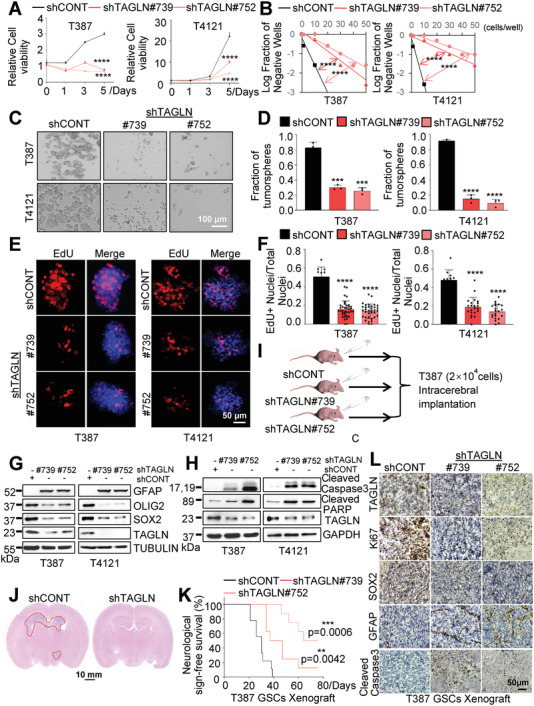
Targeting TAGLN impairs GSC self‐renewal and tumorigenicity. A) Cell viability assay of GSCs infected with the two shRNAs against TAGLN (Two Way ANOVA). B) In the limiting dilution assays, the sphere‐forming frequency was examined following TAGLN knockdown (ELDA). C,D) Representative images of neurospheres derived from GSCs expressing shCONT or C) shTAGLN and D) quantification. Scale bar, 100 µm. E) Representative images of the EdU assay after TAGLN knockdown and F) the ratio of EdU‐positive cells was calculated. Scale bar, 50 µm. G) Expression of the indicated proteins during TAGLN targeting was detected by IB. H) IB of apoptotic proteins in GSCs after TAGLN knockdown. I) Experimental design. J) Representative images of HE staining in the brains of tumor‐bearing mice. The scale bar is 10 mm. K) Kaplan‐Meier curves of mice were drawn to determine the burden of tumor progression on GSCs expressing shCONT or shTAGLN (log‐rank test, *n =* 9 for each group). L) Representative IHC images of the indicated proteins stained on frozen sections of the GBM xenografts. The bar represents 50 µm. Values in (A, B, and D) represent the mean ± SD from three independent experiments (**p < 0.01, ***p < 0.001, and ****p < 0.0001).

To elucidate TAGLN function in GSCs, we performed loss‐of‐function studies by transducing patient‐derived GSCs with either a non‐targeting control shRNA sequence (sh*CONT*) or one of two distinct short hairpin RNAs (sh*TAGLN*#739 and sh*TAGLN*#752) to knock down TAGLN expression. We confirmed that both shTAGLNs reduced TAGLN expression compared with shCONT (Figure S4A,B, Supporting Information). Targeting TAGLN attenuated the viability of patient‐derived GSCs (**Figure** **4A**; Figure S4C, Supporting Information). Sphere formation is a surrogate marker of self‐renewal, albeit with caveats. Following TAGLN knockdown in GSCs, the tumorsphere formation frequency of GSCs was reduced, as assayed by in vitro limiting dilution (Figure 4B; Figure S4D, Supporting Information). Stable TAGLN knockdown reduced the ability of GSCs to form neurospheres (Figure 4C,D; Figure S4,F, Supporting Information). TAGLN silencing also inhibited DNA replication, as revealed by 5‐Ethynyl‐2‐deoxyuridine (EdU) incorporation (Figure 4E,F; Figure S4G,H, Supporting Information). Decreased levels of GSC markers SOX2 and OLIG2 were observed in TAGLN‐targeted GSCs compared with controls, accompanied by increased expression of GFAP (Figure 4G; Figure S4I, Supporting Information). Furthermore, TAGLN silencing induced apoptosis measured by elevated Cleaved Caspase3 and Cleaved polyadenosine 5’‐diphosphate‐ribose polymerase (PARP) by IB (Figure 4H; Figure S4J, Supporting Information). These data indicated that targeting TAGLN disrupted GSC maintenance.

In vivo tumor initiation is the gold standard for cancer stem cells. To assess GSC dependency on TAGLN in vivo, 2 × 10^4^ T387 GSCs transduced with lentiviruses encoding either shTAGLN or shCONT were implanted intracranially into the frontal lobes of immunocompromised mice. Tumor‐bearing brains were harvested after the presentation of the first neurological sign in any group (Figure 4I). Consistent with our findings in vitro, no tumor was found in animals bearing GSCs transduced with shTAGLN, which prolonged tumor latency and extended the survival of tumor‐bearing mice (Figure 4J,K). Analysis of tumor‐bearing brains revealed that targeting TAGLN expression in GSCs reduced the expression levels of TAGLN, the proliferation marker Ki67, and the GSC marker SOX2, but increased levels of the GFAP differentiation marker and Cleaved Caspase3 (Figure 4L; Figure S4K, Supporting Information). Collectively, our data indicate that TAGLN is a critical GSC dependency.

### 2.5 TAGLN‐HDAC2 Promotes GSC Self‐Renewal by Regulating p53 Deacetylation

**Figure 5:**
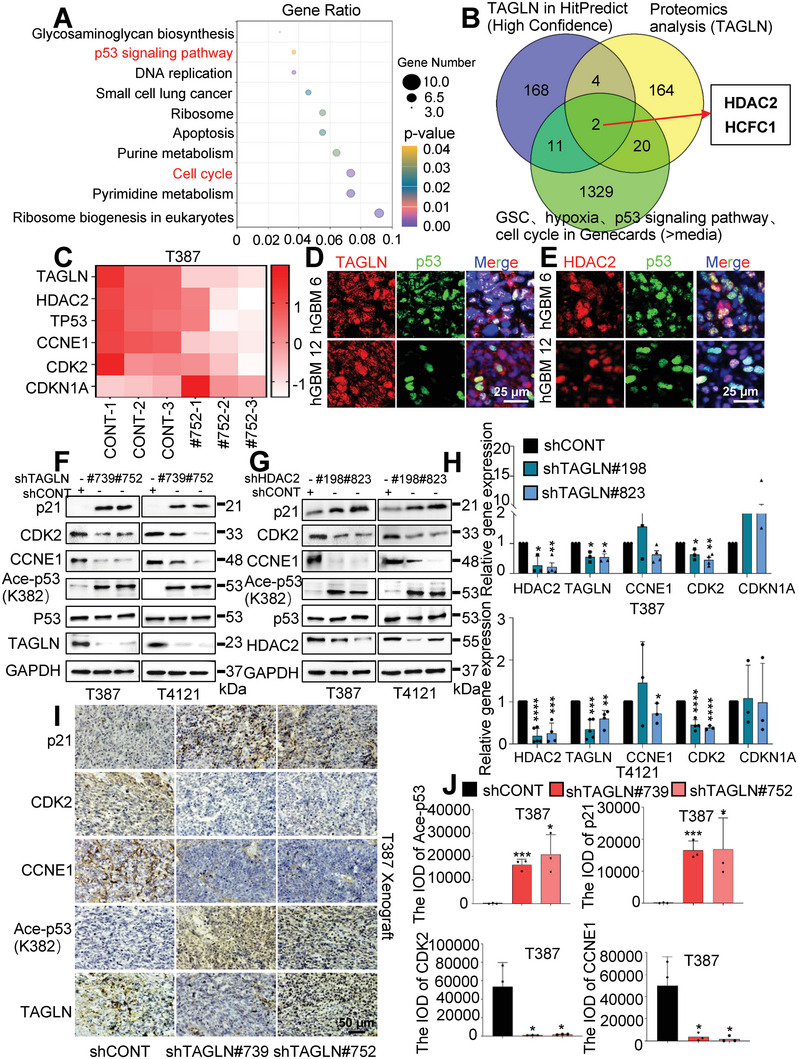
The preservation of the self‐renewal capacity of GSCs benefits from the activation of the TAGLN/HDAC2 deacetylated p53 signaling. A) Scatter plot of KEGG pathway enrichment analysis of differentially expressed genes in the RNA‐seq data after *TAGLN* knockdown. B) Proteins interacting with TAGLN were analyzed in HitPredict (high confidence, *n =* 185), proteins co‐precipitated with TAGLN were detected by mass spectrometry (*n =* 190), and proteins associated with glioma stem cells, hypoxia, p53 signaling pathway, and cell cycle in Genecards (> median, *n =* 1362), and overlapping proteins HDAC2 and HCFC1 were identified using Venn diagram. C) Heat map showing the differentially expressed genes after *TAGLN* knockdown. D) IF staining of TAGLN and p53 in hGBM tissues. Scale bar, 25 µm. E) Expression of HDAC2 and p53 in hGBM tissues was observed by IF. Scale bar, 25 µm. F,G) The indicated protein levels were examined in cell lysates of GSCs expressing shTAGLN, shHDAC2, or shCONT. H) mRNA levels of *TAGLN* and *TAGLN* target genes were assessed by qRT‐PCR following *HDAC2* knockdown. I) IHC staining of TAGLN and p53 signaling elements in GBM xenografts with TAGLN knockdown. Scale bar, 50 µm. J) Quantification of the indicated protein intensities in IHC by measuring IOD (I). Values in (H and J) represent the mean ± SD from three independent experiments (*p < 0.05, **p < 0.01, ***p < 0.001, and ****p < 0.0001).

To ascertain the mechanism by which TAGLN maintains GSC self‐renewal, we performed comparative RNA‐seq on GSCs transduced with shTAGLN or shCONT. *TAGLN* silencing induced extensive changes in GSC gene expression (Figure S5A, Supporting Information). Concordant with phenotypic effects of targeting *TAGLN*, the largest subset of downregulated genes was primarily involved in cell cycle control and the p53 signaling pathway (**Figure** **5A**). Gene set enrichment analysis (GSEA) of data from TCGA and CGGA in Gliovis revealed that high *TAGLN* expression correlated with the p53 signaling pathway (Figure S5B,C, Supporting Information). To determine the direct molecular connections, we immunoprecipitated TAGLN and resolved proteins that coprecipitated with TAGLN using mass spectrometry (MS). Two genes (HDAC2 and HCFC1) satisfied three criteria: 1) proteins detected in TAGLN‐IP MS but not control IP MS; 2) proteins predicted to interact with TAGLN with high confidence using HitPredict (http://www.hitpredict.org/); and 3) genes within Genecards (https://www.genecards.org/) associated with hypoxia, GSCs, p53 signaling and cell cycle regulation, displayed on a Venn diagram (Figure 5B). We prioritized *HDAC2* because of its strong association with the p53 signaling pathway and cell cycle in analysis of TCGA GBM/LGG (IDH‐WT) and CGGA GBM (IDH‐WT) datasets (Figure S5D–G, Supporting Information), relative to HCFC1 (Figure S5H–K, Supporting Information). HDAC2 contributes to tumor growth in part by controlling the deacetylation of p53 and transcriptional repression of TP53 target genes, while also mediating p53 stability.^[^ ^21^ ^]^ We then assessed all HDAC family members that were elevated in GBM and functioned as potential oncogenes. Upon *TAGLN* knock down, *HDAC2* was specifically decreased showing the relative specificity of *TAGLN* in affecting *HDAC2* expression (Figure S5L,M, Supporting Information). Genes commonly dysregulated in cancer in the p53/cell cycle signaling pathway and *HDAC2* were displayed from the RNA‐seq data upon targeting *TAGLN* (Figure 5C). GSEA analysis validated the association with *HDAC2* (Figure S5D–G, Supporting Information), indicating that the activated TAGLN/HDAC2‐p53 axis may stimulate GSC proliferation under hypoxia. *TAGLN* and *HDAC2* were strongly correlated with *TP53* and the cell cycle‐related genes *CCNE1*, *CDK2*, and *CDKN1A* in the independent CGGA (IDH‐WT) and TCGA GBM/LGG (IDH‐WT) databases (Figure S5N,O, Supporting Information). In GBM patient‐derived biopsies, TAGLN and HDAC2 each co‐localized with p53 on IF (Figure 5D,E; Figure S5P, Supporting Information). TAGLN or HDAC2 knockdown increased acetylated p53 (ace‐p53) at K382 in GSCs as measured by IB, with no perceptible alteration in total p53 or phosphorated p53 protein levels (Figure 5F and G; Figure S5Q–S, Supporting Information). Targeting TAGLN or HDAC2 in GSCs induced ace‐p53 and p21 (inhibitors of the CCNE/CDK2 complex) while reducing CCNE1 (G1 phase cyclin) and CDK2 (cyclin‐dependent kinase from G1 phase to S phase) (Figure 5F–H; Figure S5T,U, Supporting Information), but did not cause major changes in the expression of p27, CCND1, CDK4, or CDK6 (Figure S5R,S, Supporting Information). In vivo, histopathological analysis of xenografts derived from GSCs transduced with shTAGLN or shCONT revealed loss of TAGLN was associated with induction of ace‐p53 and p21 with reduced CCNE1 and CDK2 (Figure 5I,J; Figure S5V,W, Supporting Information). In gain‐of‐function studies, TAGLN overexpression in GSCs induced alterations of TAGLN/HDAC2‐deacetylated p53‐cell cycle target genes expression, opposite to that observed after TAGLN or HDAC2 knockdown, as expected (Figure S5X,Y, Supporting Information). Thus, TAGLN‐HDAC2 regulates the GSC phenotype in association with p53 regulation and cell cycle control.

### 2.6 TAGLN Binds to HDAC2 and Mediates its Degradation Primarily Through the Lysosome

**Figure 6:**
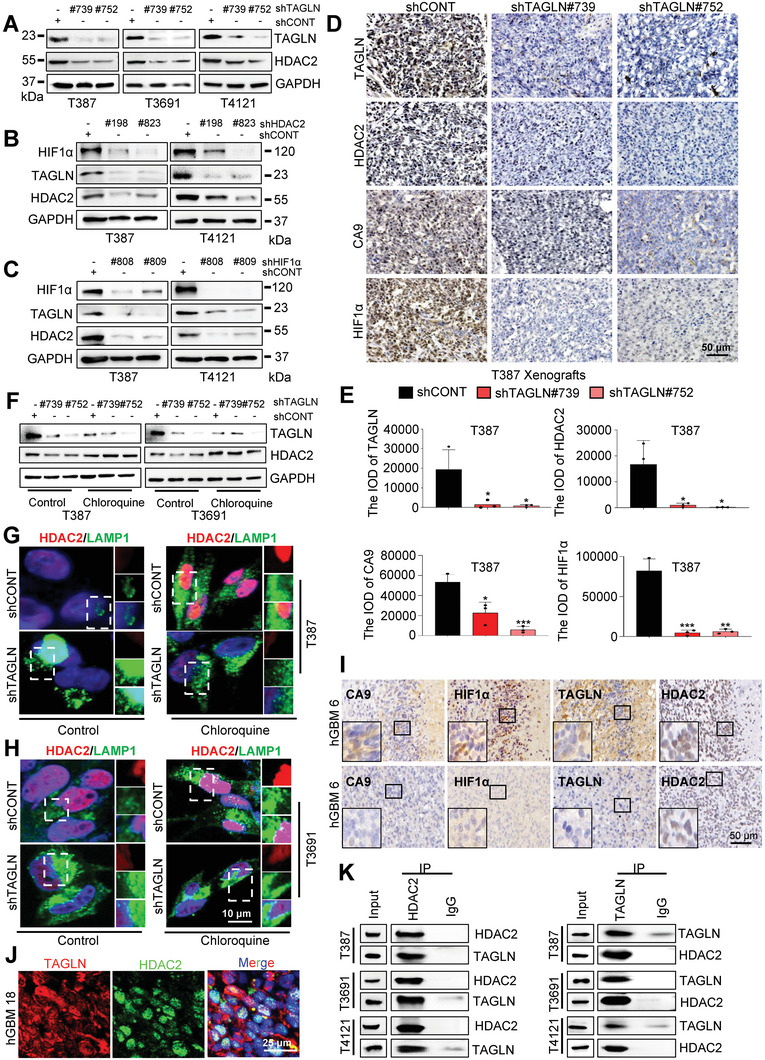
TAGLN binds to HDAC2 to form a co‐transcriptional activator complex. A) Expression of HDAC2 and TAGLN was detected by IB. B) Expression of TAGLN and HDAC2 in GSCs after HDAC2 knockdown was examined by IB. C) Expression of HDAC2, TAGLN, and HIF1α during HIF1α targeting was detected by IB under hypoxia. D) Representative IHC images of TAGLN, HDAC2, CA9, and HIF1α expression in the xenografts. Scale bar, 50 µm. E) IOD of HDAC2, TAGLN, CA9, and HIF1α were measured in (D). F) IB analysis of TAGLN and HDAC2 in different treatment groups. G,H) IF staining of HDAC2 (red) and LAMP1 (lysosomal marker; green) after DMSO or chloroquine treatment of TAGLN targeted GSCs. Scale bar, 10 µm. I) IHC staining revealed HDAC2, HIF1α, CA9, and TAGLN expression in pseudopalisades (upper) or other areas (lower) of GBM. Scale bar,50 µm. J) Representative images of TAGLN and HDAC2 expression in GBM specimens. Scale bar, 25 µm. K) Co‐IP analysis of TAGLN and HDAC2 in three GSC cell lines. Values in (E) represent the mean ± SD from three independent experiments (*p < 0.05, **p < 0.01, and ***p < 0.001).

As the TAGLN interactome revealed binding to HDAC2 localized to the nucleus (Figure 5B; Figure S5L,M, Supporting Information), we analyzed the CGGA GBM (IDH‐WT) dataset, revealing that *HDAC2* associated with poor prognosis in GBM (Figure S6A, Supporting Information). To define the interplay among TAGLN, HDAC2, and HIF1α, we first targeted TAGLN and detected a reduction in TAGLN, HDAC2, and HIF1α expression (**Figures** **6A** and 3E; Figure S6B, Supporting Information). Reciprocally, HDAC2 knockdown also reduced the levels of HDAC2, TAGLN, and HIF1α (Figure 6B). We selectively knocked down HIF1α expression and detected decreased TAGLN and HDAC2 by IB (Figure 6C), indicating an interdependent relationship among TAGLN, HDAC2, and HIF1α. Consistent with the in vitro observations, xenografts derived from GSCs transduced with shTAGLN expressed decreased HDAC2, CA9, and HIF1α protein levels measured by IHC relative to GSCs transduced with shCONT (Figure 6D,E).

The correlation between TAGLN and HDAC2 expression in GBM cells promoted the investigation of molecular mechanisms underlying the effects of TAGLN on HDAC2 expression. GSEA revealed that *TAGLN*‐correlated genes were enriched in the lysosomal pathway (Figure S6C, Supporting Information). TAGLN‐knockdown GSCs were incubated with chloroquine (which has multiple mechanisms of action, including interference of lysosomal activity and autophagy) or MG132 (a proteasome inhibitor); chloroquine restored HDAC2 downregulation in GSCs (Figure 6F–H; Figure S6D,E, Supporting Information), whereas treatment with MG132 did not affect HDAC2 stability in T387 GSCs but had an effect on T3691 GSCs (Figure S6F, Supporting Information), suggesting that lysosome‐mediated protein degradation involved in HDAC2 depletion upon TAGLN targeting. To investigate the mechanism by which HDAC2 regulates TAGLN expression, we treated HDAC2‐knockdown GSCs with the proteasome inhibitor MG132 or the lysosomal inhibitor chloroquine for 2 h and used the same dose of DMSO as a control. As a result, MG132 increased the expression of TAGLN, while chloroquine caused no noticeable change (Figure S6G,H, Supporting Information). Furthermore, the silencing of HDAC2 by two independent shRNAs enhanced TAGLN ubiquitylation in T387 and T4121 GSCs (Figure S6I, Supporting Information). Overall, these results suggest that TAGLN mediates HDAC2 degradation primarily through the lysosome, while silencing HDAC2 in GSCs disrupts the stabilization of TAGLN, leading to its proteasomal degradation.

To explore TAGLN and HDAC2 expression in GBM patient surgical specimens, we performed IHC staining and observed that HDAC2 (which is a hypoxia‐related gene), TAGLN, CA9 (a hypoxic marker), and HIF1α were all located in GBM tumor cell nuclei and highly expressed in pseudopalisades (upper) (Figure 6I; Figure S6J,K, Supporting Information). IF analysis confirmed TAGLN and HDAC2 (positively related to the GSC markers) localization (Figure 6J; Figure S6L,M, Supporting Information). In the CGGA (IDH‐WT) and TCGA GBM/LGG (IDH WT) datasets, *TAGLN* and *HDAC2* mRNA levels were positively correlated (Figure S5N,O, Supporting Information) and patients whose tumors were *TAGLN* ^hi^ *HDAC2* ^hi^ had a worse prognosis (Figure S6N,O, Supporting Information). Based on the co‐expression, we hypothesized that TAGLN and HDAC2 directly interacted. Indeed, Co‐IP revealed that TAGLN coprecipitated with HDAC2. Reciprocally, HDAC2 coprecipitated with TAGLN in the same lysates, suggesting that TAGLN and HDAC2 formed a transcriptional regulator complex that sustained each other's stability and promoted GSC proliferation (Figure 6K). To further elucidate the interaction between HIF1α, TAGLN, and HDAC2, we employed Co‐IP in normoxia and hypoxia. Our data observed that HIF1α combined with the TAGLN/HDAC2 complex under hypoxia, while the binding of HIF1α or TAGLN to HDAC2 was not found under normoxia (Figure S6P, Supporting Information). Taken together, our data suggest that HIF1α, TAGLN, and HDAC2 are mutually regulated, forming complexes that maintain the self‐renewal of GSCs under hypoxia.

### 2.7 Targeting TAGLN/HDAC2 with Sodium Valproate and Natural Borneol Combination Therapy

**Figure 7:**
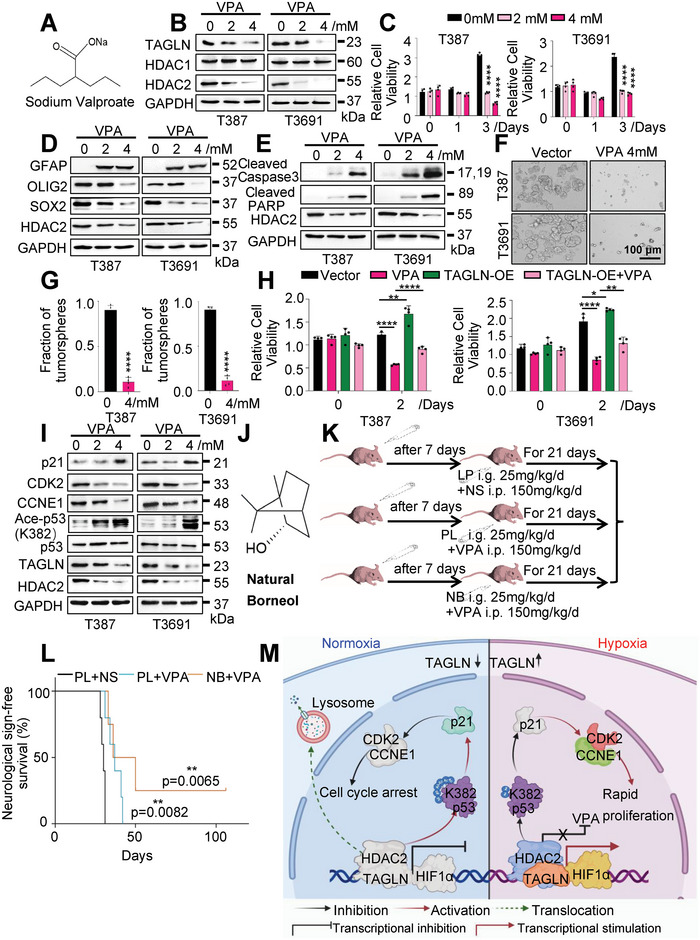
Sodium valproate blocks TAGLN/HDAC2 to promote P53 acetylation and the self‐renewal of GSCs. A) Schematic representation of the structural formula of sodium valproate. B) T387 and T3691 GSCs were treated with 2 or 4 mM sodium valproate for 48 h in hypoxia, and the levels of TAGLN, HDAC1, and HDAC2 were analyzed by IB. C) Viability of GSCs over a 3‐day period following sodium valproate treatment (Two Way ANOVA). D) GSCs’ markers expression detected by IB in sodium valproate‐treated GSCs. E) Expression of apoptotic proteins in GSCs was analyzed following treatment with sodium valproate. F) Representative images and G) quantification of neurosphere formation in GSCs treated with sodium valproate (Student's t‐test). Scale bar, 100 µm. H) Cell viability assays after sodium valproate or normal saline treatment in GSCs with or without TAGLN overexpression (Student's t‐test). I) p53 signaling elements were assessed following sodium valproate treatment. J) Schematic of the structural formula of natural borneol. K) Experimental design. L) Kaplan‐Meier curves of tumor‐bearing mice treated with the indicated treatments (Log‐rank test, *n =* 5 for each group). M) Mechanistic model diagram of TAGLN regulating GSC stability. Under normoxia, TAGLN remained at a low level, and the transcriptional activity of HDAC2, which interacted with TAGLN, was reduced and degradation was enhanced, resulting in a decrease in the self‐renewal of GSC. Hypoxia can induce upregulation of TAGLN expression, by forming a transcriptional complex with HIF1α and upregulating the level of deacetylation of p53 after binding to HDAC2, thereby promoting cell cycle progression and maintaining the stability of GSC. Values in (C, G, and H) represent the mean ± SD from three independent experiments (*p < 0.05, **p < 0.01, and ****p < 0.0001).

Based on the GSC dependency on TAGLN in vivo, we sought clinically efficacious small molecule inhibitors that selectively target TAGLN/HDAC2 activity for GBM therapy. Sodium valproate (VPA; structure shown in **Figure** **7A**) is an antiepileptic drug, which can selectively inhibit the activities of HDAC1 and HDAC2.^[^ ^22^ ^]^ Sodium valproate treatment inhibits GSC proliferation and survival, as well as promotes GSC differentiation.^[^ ^23^ ^]^ However, the exact mechanism of its killing effect on GSCs as an HDAC2 inhibitor remains to be elucidated. To confirm the therapeutic effects of sodium valproate on GSCs, we treated GSCs with varying concentrations of sodium valproate and found that the protein levels of HDAC2, but not HDAC1, was attenuated at 4 mM, which was accompanied by decreased TAGLN expression (Figure 7B; Figure S7A, Supporting Information). Under hypoxia, we applied VPA to GSCs, and Co‐IP was performed to verify the protein interaction between TAGLN and HDAC2. Treatment of T387 and T4121 GSCs with 4mM VPA significantly weakened the ability of TAGLN to bind to HDAC2 (Figure S7B, Supporting Information). Similar to results from genetic targeting of TAGLN, GSC cell viability was reduced with increasing concentrations of sodium valproate treatment (Figure 7C), with reduced GSC marker expression levels and induction of GSC differentiation and apoptosis (Figure 7D,E). Sodium valproate treatment inhibited GSC tumorsphere formation (Figure 7F,G). As valproate has been considered an HDAC2 inhibitor, we investigated the role of TAGLN in tumor cell response by rescuing treatment effects with TAGLN overexpression (TAGLN‐OE) in GSCs. TAGLN‐OE partially rescued GSC self‐renewal following sodium valproate treatment (Figure 7H). Likewise, sodium valproate suppressed the activity of the TAGLN‐HDAC2‐p53 axis in GSCs (Figure 7I), which TAGLN‐OE partially rescued in (Figure S7C, Supporting Information). Thus, valproate targets TAGLN/HDAC2 to inhibit GSCs, suggesting that blocking TAGLN/HDAC2 may result in a high therapeutic index for GBM treatment.

Many potentially effective agents against GBM in vitro fail in vivo due to constraints of the blood‐tumor barrier (BTB). To investigate whether sodium valproate could suppress xenograft growth in vivo, we randomized tumor‐bearing mice after 14 days into groups intraperitoneally injected with normal saline (NS) as the vehicle control or different concentrations of sodium valproate for an additional 14 days. Preliminary experiments did not yield differences in survival of tumor‐bearing mice and the lack of changes in HDAC2 and TAGLN expression among treatment groups as a pharmacodynamic study suggested that ineffective drug concentrations were achieved (Figure S7D,E, Supporting Information). Considering the systemic toxicity and drug delivery efficiency of sodium valproate, we initiated treatment earlier (day 7) of tumor‐bearing mice with sodium valproate at 150 mg kg^−1^ for 14 days and found extension of tumor latency, but did not achieve an ideal therapeutic effect (Figure S7F, Supporting Information). Based on the limitations in drug delivery as a limitation in brain tumor treatment, we considered options to improve valproate efficacy. Natural borneol improves the delivery efficiency of chemotherapeutic drugs by increasing the permeability of the BTB^[^ ^24^ ^]^(Figure 7J). To assess the combinatorial antitumor effects of valproate and natural borneol in vivo, we initiated treatment of GSC‐derived xenografts one week after tumor formation with borneol (25 mg kg^−1^) or paraffin liquid (PL) control administered by gavage; 30 minutes later, mice were treated with sodium valproate (150 mg kg^−1^) or NS (Figure 7K). The combination of natural borneol and sodium valproate prolonged the survival of tumor‐bearing mice and increased the VPA levels in xenografts compared with sodium valproate monotherapy (Figure 7L; Figure S7G, Supporting Information). Compared to NS control or sodium valproate monotherapy, the combination of natural borneol and sodium valproate decreased TAGLN, HDAC2, CCNE1, CDK2, and Ki67 while increasing ace‐p53, p21, and Cleaved Caspase3, but did not affect the expression of p53, CDK4, CCND1, CDK6, p27, and P‐p53 (S392) (Figure S7H,I, Supporting Information). Collectively, sodium valproate represents a potentially useful GBM therapy due to targeting HDAC2‐TAGLN‐p53 with improved efficacy through combination with borneol.

## 3 Discussion

GBM remains lethal and incurable despite maximal therapy. GBMs have been extensively characterized genetically, but precision oncology has offered little benefit in neuro‐oncology. One contributing factor to GBM treatment failure is likely cellular heterogeneity, including the presence of self‐renewing GSCs that commonly resist conventional therapy and augment angiogenesis, invasion, and immune escape. Here, we identified TAGLN as a novel target in GSCs that serves to bridge histone deacetylase function and p53 to reveal improved therapeutic targeting. TAGLN is preferentially expressed by GSCs, suggesting a potentially high therapeutic index. In contrast to previous reports that TAGLN serves as a suppressor during tumorigenesis initiation in other cancers^[^ ^12^, ^13^, ^25^ ^]^, TAGLN acts as a functional marker for tumor stemness in GBM.

Hypoxia promotes tumor growth and therapeutic resistance.^[^ ^8^, ^26^ ^]^ The expression of TAGLN, a gene involved in hypoxic immunity, predicts a poor prognosis of gastric cancer.^[^ ^27^ ^]^ HIFs promote GSC self‐renewal and tumor angiogenesis.^[^ ^9^, ^28^ ^]^ HIF1α, but not HIF2α, regulated hypoxic induction of TAGLN expression (Figure 3A,B,G; Figure S3B, Supporting Information). As inhibition of TAGLN reduced HIF1α transcription and translation (Figure 3H,E), TAGLN may also function as a transcriptional regulator. Although TAGLN does not have a nuclear localization sequence, it binds to transcription factors and enters the nucleus. As predicted, TAGLN bound at the binding motif predicted by ChIP‐seq on the HIF1α promoter (Figure 3I–K). To the best of our knowledge, the present study is the first to show a hypoxic transcription complex of TAGLN‐HIF1α in GSCs.

Dysregulation of TAGLN contributes to tumor growth.^[^ ^13^, ^29^ ^]^ In this study, we found that TAGLN was critical to maintain GSCs (Figure 4). We found interactions between TAGLN and the p53 axis contributing to GSC proliferation and survival. p53 and PTEN regulate TAGLN to inhibit bladder cancer cell proliferation.^[^ ^12^ ^]^ As a result, we now broaden the molecular interactome with TAGLN to add HDAC2 regulating p53 deacetylation at K382, in turn, mediating acceleration of cell cycle progression. HDAC2 is involved in p53 deacetylation to prevent proliferation arrest in tumor cells.^[^ ^30^ ^]^

Genetic targeting of TAGLN reduced in vivo tumor growth in GBM, resulting in an increase in survival of tumor‐bearing mice, supporting TAGLN as an effective target (Figure 4K). Direct TAGLN inhibitors have not been reported, whereas effective drugs that block the TAGLN/HDAC2‐p53 axis offer the possibility of therapy for GBM patients. Clinical retrospective studies have shown that the antiepileptic drug sodium valproate, alone or in combination with radiotherapy or chemotherapy, can suppress seizure activity and extend the survival of GBM patients^[^ ^23^, ^31^ ^]^, which may be explained by its additional pharmacodynamic properties, particularly its HDAC2 inhibition. Although prior studies have shown that sodium valproate is an effective glioma treatment^[^ ^32^ ^]^, the mechanism by which sodium valproate inhibits the growth of GSCs remains unclear. Sodium valproate inhibited GSC proliferation, phenocopying silencing TAGLN/HDAC2, suggesting the specificity of sodium valproate inhibiting TAGLN/HDAC2.

GBMs display regionally intact BTB^[^ ^33^ ^]^, which hampers drug penetration, accumulation, and uptake in GBM tumors, limiting chemotherapeutic efficacy. As an anti‐epileptic drug, valproate is brain penetrant but the concentrations may be insufficient to reach therapeutic concentrations in the tumor, but poses toxicity to normal tissues. Natural borneol can open the blood‐brain barrier and enhance the penetration of drugs into the brain.^[^ ^24^, ^34^ ^]^ Wang and co‐workers confirmed that natural borneol monotherapy induced GBM cell apoptosis by regulating HIF1α expression.^[^ ^35^ ^]^ Here, we demonstrate that sodium valproate combined with natural borneol prolonged the survival of tumor‐bearing mice and increased the VPA levels in xenografts (Figure 7L; Figure S7G, Supporting Information).

In conclusion, our study provides a novel insight into TAGLN/HDAC2‐promotion of cell cycle progression through deacetylation of p53 at K382 under hypoxia and offers a targeted approach to improve the therapeutic effect of GBM. TAGLN may be an independent predictor of overall survival in GBM and hypoxic transcriptional regulators. The combination of TAGLN/HDAC2 blocker and natural borneol for treating GBM patients reveals that combined traditional Chinese and Western medicine enhance the delivery of sodium valproate into GBM tumors and improve preclinical efficacy. These results support the development of TAGLN/HDAC2 blockers as anti‐GSC treatments and open the possibility of generalizing these findings to the therapy of other malignant tumors.

## 4 Experimental Section

### Cell Lines and Cell Culture

All cell lines were dissociated and sorted from GBM patient surgical samples and xenografts, as previously described.^[^ ^36^ ^]^ All GSC and NPC cell lines were generously gifted by Dr. Shideng Bao in the United States. GSCs and NPCs were cultured in Neurobasal‐A medium supplemented with 10% B27 (Cat# 354277, Gibco), 20 ng mL^−1^ EGF (Cat# 105‐04, PrimeGene), 20 ng mL^−1^ bFGF (Cat# 104‐02, PrimeGene), 1 mM sodium pyruvate (Cat# 11360070, Gibco), 2 mM l‐glutamine (Cat# 25030081, Gibco), and 100 U mL^−1^ penicillin‐streptomycin (Cat# 15140‐122, Gibco). Differentiated GSCs (DGCs), 293T (ATCC), and glioma cells were cultured in DMEM supplemented with 10% FBS (Cat# A3161001C, Gibco). Cells were cultured in a humidified 37 °C, 5%CO_2_, 21%, or 1% O_2_ incubator for no >2 months after recovery. Moreover, all cell lines were shown to be free of mycoplasma infection (Cat# C0297S, Beyotime Biotechnology).

### Human GBM tissues

This study was approved by the Medical Ethics Committee, Tongji Medical College, Huazhong University of Science and Technology, Wuhan, China (serial no. [2021] IEC (A244)). Samples were collected from each patient after obtaining their written informed consent. All the patient studies were conducted in accordance with the principles of the Declaration of Helsinki. The clinical information of the patients is detailed in Table S1 (Supporting Information).

### Intracranial Tumor Assay and Medicine Treatment

Healthy male and female nude BALB/c mice were purchased from the Beijing Vital River Laboratory Animal Technology Co., Ltd (Beijing, China) and were housed in peculiar pathogen‐free cages. Five nude mice in each cage were checked daily by certified veterinarians and laboratory personnel and minimized all discomfort and pain in mice. Nude mice were randomly divided into groups for all experiments. GSCs were transduced with shCONT or shTAGLN by lentiviral infection for 48 h. The GSC suspension (2 × 10^4^ cells for transducing GSC T387) was intracranially injected into the brains of 4‐5‐week‐old nude mice as described previously. For the treatment experiments, tumor‐bearing mice were gavaged with natural borneol (25 mg kg^−1^, i.g., China Institute for the Control of Pharmaceutical and Biological Products) or paraffin liquid (25 mg kg^−1^, i.g., Cat# 8042‐47‐5, Aladdin) on day 7, half an hour later, intraperitoneally injected with sodium valproate (150 or 300 mg kg^−1^, i.p., Cat# HY‐10585A, MCE) or normal saline for 14 or 21 days. To compare the growth of the xenografts between the different groups, the brain tissue of the mice in the experimental group was harvested simultaneously when the control group mice developed neurological symptoms and performed with H&E staining. Mice were anesthetized by intraperitoneal injection of a mixture of ketamine (100 mg kg^−1^) and xylazine (10 mg kg^−1^) before all surgical procedures. After surgery, the mice were euthanized by cervical dislocation under deep anesthesia. All mouse studies were performed using protocols approved by the Experimental Animal Ethics Committee, Huazhong University of Science and Technology (serial no. S1819).

### Plasmid Generation, Lentiviral Transfection, and Reagent Treatment

TAGLN overexpression plasmid was generated by cloning the coding sequence (CDS) of the human TAGLN gene into the pLVX‐puro‐3 × FLAG vector (Cat# 632164, Clontech). The shRNA sequence was shown in Table S2 (Supporting Information) and shRNAs for *HIF1A*, *EPAS1*, *TAGLN*, and *HDAC2* were generated from pLKO.1‐puro (Cat# SHC002, Sigma‐Aldrich). Packaging vectors *psPAX2* (5 µg) (RRID: Addgene_12260, Addgene), and *p‐CMV‐VSV‐G* (5µg) (Cat# 8454, Addgene) were co‐transfected with lentiviral vectors expressing non‐target control shRNA, specific shRNA, or full‐length *TAGLN* (5µg) into 293T cells for lentivirus production. Lentiviral particles were obtained 48 h post‐transfection by filtering with a 0.45‐µm filter and then used to treat GSCs. 48 h after the virus infection of GSCs, the medium was changed and GSCs were treated with puromycin (2 mg mL^−1^, Cat# A1113803, Gibco) for 24 h.

For cell reagent treatments, transfected GSCs were cultured for 48 h under hypoxic conditions and treated with lysosome or the proteasome inhibitor, chloroquine (10 mM, Cat# HY‐17589A, MCE) or MG132 (10 µM, Cat# HY‐13259, MCE) for 2 h. GSCs were co‐incubated with varying concentrations of sodium valproate for the indicated time under hypoxic conditions.

### Hematoxylin‐eosin (HE), Immunohistochemistry (IHC), and Immunofluorescence (IF)

For HE and IHC staining, paraffin sections of GBM tissue or xenografts were first placed in a 62 °C oven for 1–2 h. Sections were then deparaffinized with xylene, followed by successive incubation with 100%, 95%, 75%, 50% ethanol, and finally hydration. Sections were heat‐induced antigen retrieval with sodium citrate or EDTA buffer according to the antibody instructions. Sections were washed three times with PBS and incubated with an appropriate amount of 3% H_2_O_2_ for 10 min at room temperature, washed three times with PBS, and blocked with 5% serum of the corresponding species for 1 h at room temperature. Next, the primary antibody was added and incubated overnight at 4 °C. The next day, sections were washed three times with PBS and incubated with secondary antibodies for 30 min at 37 °C, followed by DAB detection with horseradish peroxidase (HRP) conjugate and counterstaining of nuclei with hematoxylin. Quantitative analysis was performed by evaluating the mean integrated optical density using Image‐Pro Plus (IPP) software.

For IF staining, frozen GBM tissue was embedded with OCT compound and sectioned. Frozen sections were first fixed with 4% PFA for 20 min, rinsed three times with PBS, and incubated with 0.5% Triton‐X100 (Cat# T8200, Solarbio) for 20 min. Sections were rinsed three times with PBS, blocked with 5% normal donkey serum (NDS) for 1 h, and then incubated with primary antibody overnight at 4°C. The next day, after three washes with PBS, samples were performed with the Donkey anti‐Rabbit IgG (H+L) Cross‐Adsorbed Secondary Antibody, Alexa Fluor 546 (Cat# A10040, RRID:AB_2534016, Thermo Fisher Scientific), and the Donkey anti‐Mouse IgG (H+L) Cross‐Adsorbed Secondary Antibody, Alexa Fluor 488 (Cat# A21202, RRID:AB_141607, Thermo Fisher Scientific) for 1 h. After three washes in PBS, the nuclei were counterstained with DAPI (Cat# D1306, Thermo Fisher Scientific) and sealed with antifade. Images were taken with an Olympus microscope (Olympus FV1000, Japan).

IHC was performed using antibodies against anti‐TAGLN (Cat# ab14106, RRID:AB_443021, Abcam), anti‐SOX2 (Cat# ab171380, RRID:AB_2732072, Abcam), anti‐HIF1α (Cat# 66730‐1‐Ig, RRID: AB_288208, Proteintech), anti‐CA9 (Cat# NB100‐417, RRID:AB_10003398, NOVUS), anti‐GFAP (Cat# 80788, RRID:AB_2799963, Cell Signaling Technology), anti‐Ki67(Cat# 27309‐1‐AP, RRID:AB_2756525, Proteintech), anti‐Cleaved Caspase3 (Cat# 9664, RRID:AB_2070042, Cell Signaling), anti‐HDAC2 (Cat# ab219053, Abcam), anti‐Acetyl‐p53(K382) (Cat# 2525, RRID:AB_330083, Cell Signaling Technology), anti‐p21 (Cat# 2947, RRID:AB_823586, Cell Signaling Technology), anti‐CCNE1 (Cat# ET1612‐16, HUABIO), anti‐CDK2 (Cat# YT0832, Immunoway biotechnology), anti‐CCND1 (Cat# 26939‐1‐AP, RRID:AB_2880691, Proteintech), anti‐p27 (Cat# 3686, RRID:AB_2077850, Cell Signaling Technology), and anti‐CDK4 (Cat# 12790, RRID:AB_2631166, Cell Signaling Technology).

IF antibodies against rabbit anti‐TAGLN (Cat# ab14106, RRID:AB_443021, Abcam), mouse anti‐SOX2 (Cat# ab171380, RRID:AB_2732072, Abcam), mouse anti‐HIF1α (Cat# 66730‐1‐Ig, RRID: AB_288208, Proteintech), mouse anti‐CA9 (Cat# YM3076, Immnuoway), mouse anti‐OLIG2 (Cat# MABN50, RRID:AB_10807410, Millipore), mouse anti‐CD15 (Cat# 4744, RRID:AB_1264258, Cell Signaling), mouse anti‐CD133 (Cat# BF0403, RRID:AB_2833933, Affinity Biosciences), rabbit anti‐HDAC2 (Cat# ab219053, Abcam), mouse anti‐HDAC2 (Cat# sc‐9959, RRID:AB_627704, Santa Cruz), mouse anti‐LAMP1 (Cat# ab25630, RRID:AB_470708, Abcam) and mouse anti‐p53 (Cat# sc‐126, RRID:AB_628082, Santa Cruz) were used.

### Cell Viability Assay

GSCs were seeded into 96‐well plates at a density of 1000 cells per well in four replicate wells. Plates were removed on days 0, 1, 3, and 5 post‐inoculation under hypoxia. Cell viability was quantified using the CellTiter‐Glo Luminescent Assay (Cat# G9242, Promega) as previously described.^[^ ^10^ ^]^ All the data were normalized to day 0.

### Sphere Formation

Briefly, the indicated GSCs (1×10^3^ cells per well) were seeded in triplicate in 24‐well plates under hypoxic conditions, and the total number of neurospheres in each well was quantified after 3 days.

### Limiting Dilution Assay

Digested GSCs were plated at an increasing number of 1, 10, 20, 30, 40, or 50 cells per well in 96‐well plates with 6 repetitions, and each well was checked and quantified the percentage of wells with tumorspheres after 5 days under hypoxia (described in^[^ ^10^ ^]^). *p*‐values were computed by Extreme Limiting Dilution Analysis (ELDA) software available at http://bioinf.wehi.EdU.au/software/elda/.

### EdU Incorporation Analysis

EdU assays were performed by following the manufacturer's protocol as previously described.^[^ ^10^ ^]^ Spheroids were first incubated with EdU (Cat# C10310‐1, RIBOBIO) for 2 h and then fixed with 4% paraformaldehyde. The spheroids were incubated with 0.5% Triton‐X100, treated with 1 × Apollo staining reaction solution, and stained with DAPI. At least 10 GSCs neurospheres per group were examined for EdU‐positive cells.

### Quantitative Real‐Time PCR (qRT‐PCR)

Total RNA was extracted using the TRIzol Total RNA Extraction Reagent (Cat# 10606ES60, YEASEN). 1 µg RNA was reverse transcribed into cDNA using the Hifair III 1st Strand cDNA Synthesis SuperMix (Cat# 11137ES60, YEASEN). qRT‐PCR was performed using Hieff qPCR SYBR Green Master Mix (Cat# 11203ES03, YEASEN). All values were obtained in triplicate. The primers used for qRT‐PCR were designed using PrimerBank. The primers used for human samples are listed in Table S3 (Supporting Information).

### Immunoblotting (IB)

Lysed cells on ice using RIPA buffer (50 mM Tris‐HCl, pH 8.0, 150 mM NaCl, 0.5% NaCl, 1% NP‐40, 0.1% SDS) containing protease/phosphatase inhibitors for 15 min, pipetting every 5 min. The mixture was then centrifuged at 12 000 rpm for 15 min at 4 °C, the supernatant was collected and protein lysates were quantified by BCA Protein Concentration Assay Kit (Cat# BL521A, Biosharp). Lysates were boiled in 5 × protein loading buffer for 8 min and 40 µg of protein was separated on SDS‐PAGE Bis‐Tris gels and transferred to PVDF membrane (Cat# SEQ00010, EMD Millipore). After blocking the membrane with 5% skim milk for 1 h at room temperature, the designated primary antibodies were incubated overnight on the corresponding molecular weight bands at 4 °C. Specific antibodies against TAGLN (Cat# ab14106, RRID:AB_443021, Abcam), TAGLN (Cat# GTX101608, RRID: AB_1952134, GeneTEX), SOX2 (Cat# ab171380, RRID:AB_2732072, Abcam), HIF1α (Cat# 66730‐1‐Ig, RRID: AB_2882080, Proteintech), HIF2α (Cat# NB100‐122, RRID:AB_10002593, NOVUS), CA9 (Cat# NB100‐417, RRID:AB_10003398, NOVUS), GFAP (Cat# 80788, RRID:AB_2799963, Cell Signaling Technology), Cleaved Caspase3 (Cat# 9664, RRID:AB_2070042, Cell Signaling Technology), Cleaved PARP (Cat# 5625, RRID:AB_10699459, Cell Signaling Technology), HDAC2 (Cat# sc‐9959, RRID:AB_627704, Santa Cruz), HDAC1 (Cat# sc‐81598, RRID:AB_2118083, Santa Cruz), Ace‐p53(K382) (Cat# 2525, RRID:AB_330083, Cell Signaling Technology), p21 (Cat# 2947, RRID:AB_823586, Cell Signaling Technology), CCNE1 (Cat# 4129, RRID:AB_2071200, Cell Signaling Technology), CDK2 (Cat# 2546, RRID:AB_2276129, Cell Signaling Technology), CCND1 (Cat# 2978, RRID:AB_2259616, Cell Signaling Technology), p27 (Cat# 3686, RRID:AB_2077850, Cell Signaling Technology), CDK4 (Cat# 12790, RRID:AB_2077850, Cell Signaling Technology), CDK6(Cat# 3136, RRID:AB_2229289, Cell Signaling Technology), P‐p53(Ser392) (Cat# 9281, RRID:AB_331462, Cell Signaling Technology), p53 (Cat# sc‐126, RRID:AB_628082, Santa Cruz), DDDDK‐tag (Cat# AE005, RRID:AB_2770401, Abclonal), P‐p53(Ser15)(Cat# 28961‐1‐AP, RRID:AB_2881236, Proteintech), GAPDH (Cat# sc‐47724, RRID:AB_627678, Santa Cruz) and α‐Tubulin (Cat# AC012, RRID:AB_2768341, Abclonal) were used for the immunoblotting analysis.

The next day, incubated with the peroxidase‐affiniPure goat anti‐rabbit IgG(H+L) (Cat# 111‐035‐003, RRID:AB_2313567, Jackson ImmunoResearch Laboratories) or the peroxidase‐conjugated goat anti‐mouse IgG(H+L) (Cat# 115‐035‐003, RRID:AB_10015289, Jackson ImmunoResearch Laboratories). ECL chemiluminescence detection reagent (Cat# PI34080, Thermo Fisher Scientific) was added for imaging, and band analysis was performed using Image Lab Software.

### Co‐Immunoprecipitation Assay (Co‐IP)

GSCs were collected after hypoxia and lysed in Pierce IP Lysis Buffer (Cat# 87788, Thermo Fisher Scientific) supplemented with HaltTM Protease/Phosphatase Single‐Use Inhibitor Cocktail (Cat#78442, Thermo Fisher Scientific). After lysis on ice for 15 min, the supernatant was obtained by centrifugation. 20 µL protein A/G agarose (Cat# sc‐2003, RRID:AB_10201400, Santa Cruz) was washed with 0.1% Triton‐X100, PBS, and lysis buffer to remove nonspecifically bound proteins from the beads. Total protein lysate (800 µg) was incubated with 2 µg primary antibody for 1‐2 h at 4 °C. Then it was mixed with the above supernatant‐removed beads and incubated overnight at 4 °C on a vertical shaker. After four washes with PBS the next day, the binding components were eluted by boiling with 2 × protein loading buffer and an immunoblotting assay was performed.

Rabbit anti‐TAGLN (Cat# GTX101608, 1:500, GeneTEX), rabbit anti‐HDAC2 (Cat# ab219053, 1:30, Abcam), mouse anti‐HIF1α (Cat# ab1, RRID:AB_296474, 1:200; Abcam), IgG rabbit, and IgG mouse were used for IP.

### Mass Spectrometry Assay

GSCs were lysed in IP lysis buffer and the supernatant was incubated with TAGLN (Cat# GTX101608, 1:500, GeneTEX) or IgG (Cat# ab218427, Abcam). Added 20 µL of the protein A/G agarose and incubated overnight at 4 °C. The proteins were separated by SDS‐PAGE and stained using InstaBlue Protein Staining Solution (Cat# B8226, APExBIO). The visible band was cut, treated with acetonitrile (ACN) until it turned white, and then trypsinized. Peptides were examined using a Q Exactive HF‐X mass spectrometer (Thermo Fisher). The top 40 most abundant precursors were subjected to high‐energy collisional dissociation (HCD) and examined using MS/MS.

### Chromatin Immunoprecipitation (ChIP) Assay

ChIP assay was performed according to the instructions for the EZ‐Magna ChIP A/G Chromatin Immunoprecipitation Kit (Cat# 17‐10086, Millipore Sigma). In brief, hypoxia‐treated GSCs suspensions were collected and fixed with a final concentration of 1% formaldehyde (Sigma‐Aldrich) for 10 min at room temperature, terminated with glycine, and washed three times with cold PBS. The cell suspension was centrifuged and the supernatant was removed, and cell lysis buffer containing protease inhibitor cocktail II was added to lyse cytoplasmic proteins. After 15 min of lysis on ice, the mixture was centrifuged at low speed to remove the supernatant, and the pellet was resuspended in nuclear lysis buffer. After incubation on ice for 5 min, ultrasonic fragmentation of genomic DNA into fragments of ≈500—1000 bp, the mixture was centrifuged, and the supernatant was collected.

Two aliquots of the sonicated mixture were supplemented with ChIP dilution buffer to a volume of 500 µL and taken out 1% of the mixture as “input”. HIF1α or TAGLN antibody was added to one and IgG antibody to another and incubated at 4 °C for 1—2 h. Then, 20 µL of fully suspended magnetic protein A/G beads was added to each aliquot and incubated overnight on a vertical shaker at 4 °C. The next day after centrifugation, the supernatant was removed and the beads were washed sequentially with low‐salt immune complex washing buffer, high‐salt immune complex washing buffer, LiCl immune complex washing buffer, and TE buffer. After centrifugation, resuspend in 100 µL ChIP elution buffer and incubated with proteinase K for 2 h at 62 °C. After removing the magnetic beads with a magnetic stand and purifying the DNA fragments. The relative enrichment of the indicated genes was detected by qRT‐PCR.

### ChIP‐Seq And Data Processing

ChIP‐seq libraries were constructed with ChIP and sequenced using the Illumina platform with pair‐end reads of 100–300 bp. Removed the splice sequence in ChIP‐seq raw data with Cutadapt, the low‐quality clean data were taken out with Trimmomatic,^[^ ^37^ ^]^ and FastQC^[^ ^38^ ^]^ was used for quality control and filtering. Cleaned reads were mapped against the human reference genome hg38 using Bowtie2^[^ ^39^ ^]^ with default settings. Samtools software converted the SAM format to BAM. MACS2^[^ ^40^ ^]^ was performed to search the peak value of the ChIP‐seq, and input DNA was used as the control.

The irreproducible discovery rate (IDR)^[^ ^41^ ^]^ software was used to select reproducible peaks with high confidence between two duplicates for subsequent analysis. R‐package ChIPseeker^[^ ^42^ ^]^ (ENSEMBL101) was used to annotate the peaks. MEME^[^ ^43^ ^]^‐ChIP was used for motif discovery and identification of the combined peaks. Differential peak analysis of each group was performed using the MAnorm.^[^ ^44^ ^]^

### RNA‐Seq

Digested GSCs (T387) were infected with shCONT or shTAGLN lentivirus for two days. Three biological replicates were used for each sample group. Total RNA was extracted from cells using TRIzol reagent (Cat# AM9738, Thermo Fisher Scientific). A small portion of each RNA sample was subjected to qRT‐PCR analysis to determine the knockdown efficiency before RNA‐seq analysis. Library preparation and next‐generation sequencing were performed using the NovoGene software. Data processing was performed on a public server (https://cn.novogene.com/).

### LC‐MS Conditions

In the VPA assay, LC‐MS was performed using an UltiMate 3000 RS chromatograph (Thermo Fisher, USA) connected to the Q Exactive mass spectrometer (Thermo Fisher, USA). The chromatographic elution was performed on an agela venusil C18 plus column (50 × 2.1 mm, 5 µm) with a column temperature of 40 °C. Aqueous phase A was 10mM ammonium acetate, and organic phase B was methanol. The elution gradient was 0–2 min, 80% A; 2‐3.5 min, 80% A⟶5% A; 3.5‐5.8 min, 5% A; 3.5‐3.8 min, 5% A⟶80% A; 3.8‐5 min and 80% A at a flow rate of 0.4 mL min^−1^. The injection volume was 5 µL. The mass spectra was performed using an electrospray ionization source (ESI) with negative ion scanning selected reaction monitoring mode (SRM). The ESI‐MS operating parameters used in the method were set as follows: ion spray voltage of 4000 V, sheath gas 50 arb, pilot gas 15 arb, temperature 320 °C, and collision gas. After weighing the sample, 1 mL of methanol was added, vortex mixed well, and grind with grinding beads for 5 min. Centrifuged at 13 000 rpm for 10 min and collected the supernatant with 0.22 µM filter membrane filtration and collected filtrate for analysis. The chromatogram collection and integration of VPA were processed by the software Xcalibur 4.0 (Thermo Fisher), and linear regression was performed with a weighting coefficient of 1/*x* ^2^.

### Bioinformatics Analyses

The nucleotide sequence of *TAGLN* upstream 3000 was determined from UCSC (http://www.genome.ucsc.EdU/) and the binding motif of *HIF1A* was predicted by JASPAR (https://jaspar.genereg.net/). Expression, correlation, and survival of *TAGLN*, *HDAC2*, and related genes were analyzed using Timer 2.0 (http://timer.cistrome.org/), The Cancer Genome Atlas (TCGA) (https://portal.gdc.cancer.gov/), Chinese Glioma Genome Atlas (CGGA) (http://www.cgga.org.cn/), and GLIOVIS (http://gliovis.bioinfo.cnio.es/). RNA‐seq expression profiles from the GBM marginal zone and pseudopalisade region were obtained from the Ivy Glioblastoma Atlas Project (http://glioblastoma.alleninstitute.org/).

### Quantification and Statistical Analysis

All data were presented as the mean ± SD. Three biological replicates were subjected to all in vitro cell experiments. All statistical analyses were performed using GraphPad Prism software. Differential analysis of the expression profiles of the rim region and pseudopalisade necrosis obtained by RNA‐Seq was performed using the limma package in *R*. |Log_2_FC|>1 and/or *p*<0.05 were considered statistically significant. The significance of the differences between the two groups of data was assessed using the Mann‐Whitney U test for non‐parametric data and a two‐tailed unpaired Student's t‐test for parametric data. Pearson's correlation test was used to examine the relationship between variables in the tumor tissue. The significance of the Kaplan‐Meier curves was assessed using a log‐rank test comparing the different patient or mouse groups. Experimental sample numbers (*n*) and *p*‐values are shown in the figures, figure legends, and results section.

## Conflict of Interest

The authors declare no conflict of interest.

## Author Contributions

X.Y., J.N.R., and H.L. conceived and supervised the study. H.L., S.C., Y.Z., Y.L., and X.M. designed and performed the experiments. H.L., S.C., H.M., and Y.L. analyzed the data. G.L., Z.C., P.Z., L.C., P.P., H.Z., Z.C., M.D., S.C., H.M., Q.X., B.W., S.Z., K.S., F.W., D.G., and W.Z. provided the patient samples. H.L. drafted the manuscript. H.L., Y.Z., H.M., H.L., Q.W., J.N.R., and X.Y. reviewed and edited the manuscript. L.Z., F.M., J.N.R., and X.Y. revised the manuscript. All authors reviewed the manuscript.

## Supporting information

Supporting Information

## Data Availability

The data that support the findings of this study are available from the corresponding author upon reasonable request.
