# Supplementary material for: Transgelin Promotes Glioblastoma Stem Cell Hypoxic Responses and Maintenance Through p53 Acetylation
Source: Adv Sci (Weinh). 2023 Dec 12;11(7):2305620. doi: 10.1002/advs.202305620 (PMC10870072; doi:10.1002/advs.202305620)
Supplement: Supplementary file 1 — Supporting Information [file ADVS-11-2305620-s001.pdf]

ADVANCED SCIENCE
Open Access

Supporting Information

for *Adv. Sci.*, DOI 10.1002/advs.202305620

Transgelin Promotes Glioblastoma Stem Cell Hypoxic Responses and Maintenance Through p53 Acetylation

Huan Li, Chao Song, Yang Zhang, Guohao Liu, Hailong Mi, Yachao Li, Zhiye Chen, Xiaoyu Ma, Po Zhang, Lidong Cheng, Peng Peng, Hongtao Zhu, Zirong Chen, Minhai Dong, Sui Chen, Hao Meng, QunGen Xiao, Honglian Li, Qiulian Wu, Baofeng Wang, Suojun Zhang, Kai Shu, Feng Wan, Dongsheng Guo, Wenchao Zhou, Lin Zhou, Feng Mao, Jeremy N. Rich* and Xingjiang Yu**

ADVANCED SCIENCE
Open Access

Supporting Information

for *Adv. Sci.*, DOI 10.1002/advs.202305620

Transgelin Promotes Glioblastoma Stem Cell Hypoxic Responses and Maintenance Through p53 Acetylation

Huan Li, Chao Song, Yang Zhang, Guohao Liu, Hailong Mi, Yachao Li, Zhiye Chen, Xiaoyu Ma, Po Zhang, Lidong Cheng, Peng Peng, Hongtao Zhu, Zirong Chen, Minhai Dong, Sui Chen, Hao Meng, QunGen Xiao, Honglian Li, Qiulian Wu, Baofeng Wang, Suojun Zhang, Kai Shu, Feng Wan, Dongsheng Guo, Wenchao Zhou, Lin Zhou, Feng Mao, Jeremy N. Rich* and Xingjiang Yu**

Figure S1

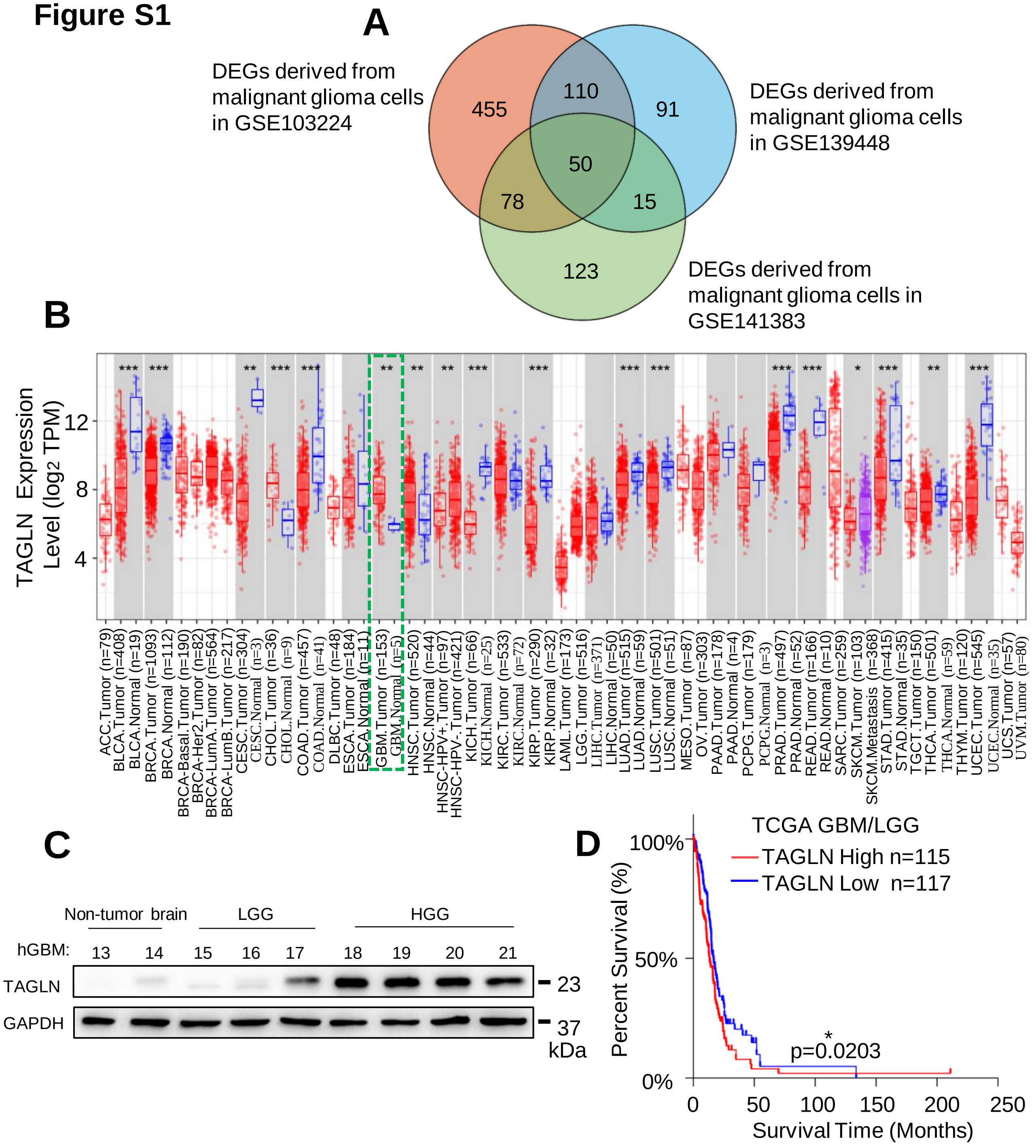

Fig S1. Increased TAGLN expression correlates with a poor prognosis in GBM.

A. The venn diagram showed the 50 genes common to the three databases in TISCH2.

B. Expression of *TAGLN* between tumor and adjacent normal tissue in all TCGA tumors. The statistical significance was computed by the Wilcoxon test.

C. Immunoblot analysis of total TAGLN expression in low-grade (LGG) or high-grade (HGG) glioma samples, showing elevated TAGLN expression in human GBM specimens.

D. Kaplan-Meier survival analysis of patients with different *TAGLN* expression levels in the Gliovis TCGA GBM/LGG (IDH-WT) dataset (Log-rank test).

Figure S2

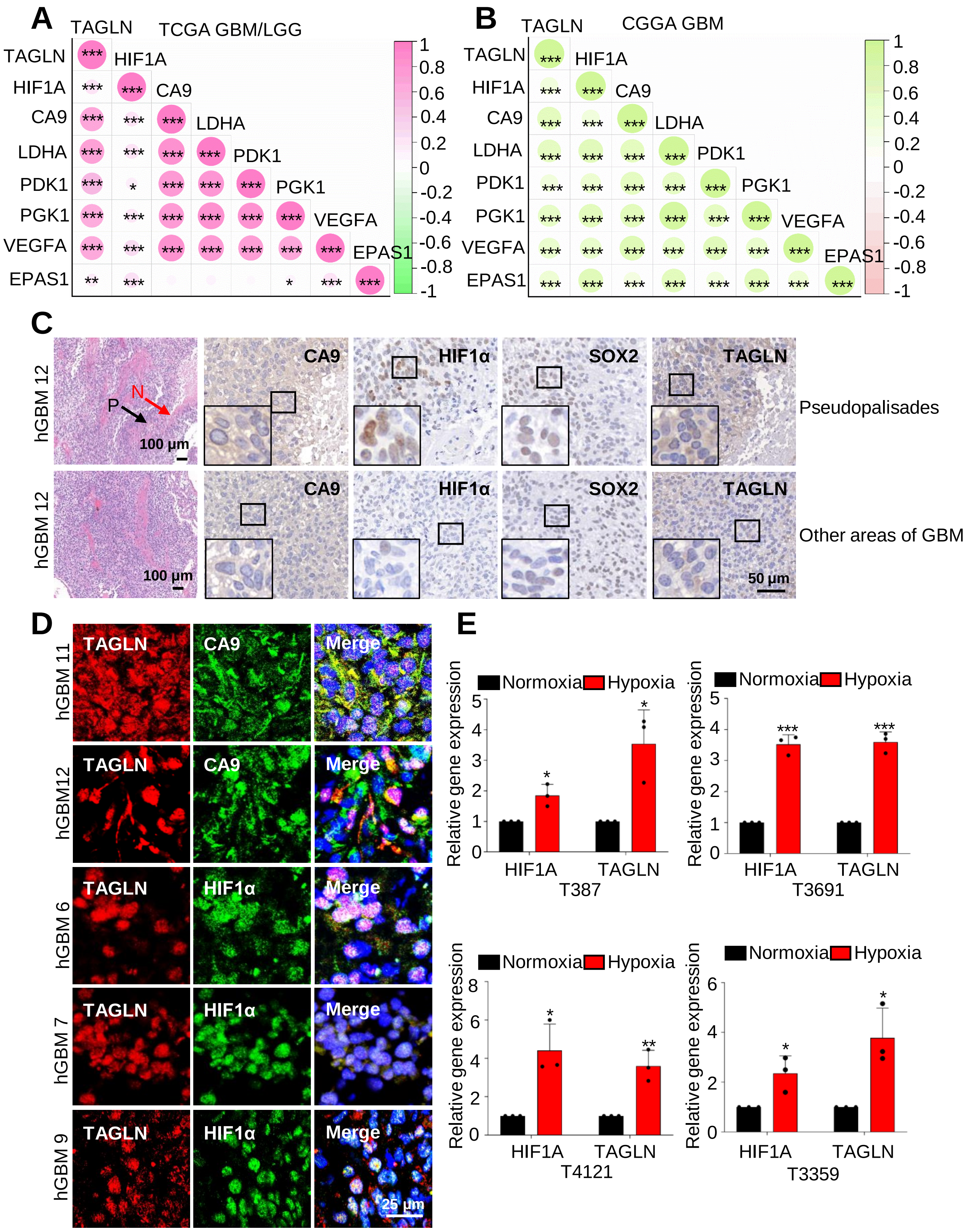

A
TCGA GBM/LGG
TAGLN
HIF1A
CA9
LDHA
PDK1
PGK1
VEGFA
EPAS1
B
CGGA GBM
C
hGBM 12
CA9
HIF1α
SOX2
TAGLN
Pseudopalisades
Other areas of GBM
100 μm
50 μm
P
N
D
hGBM 11
hGBM12
hGBM 6
hGBM 7
hGBM 9
Merge
25 μm
E
Normoxia
Hypoxia
Relative gene expression
T387
T3691
T4121
T3359

F

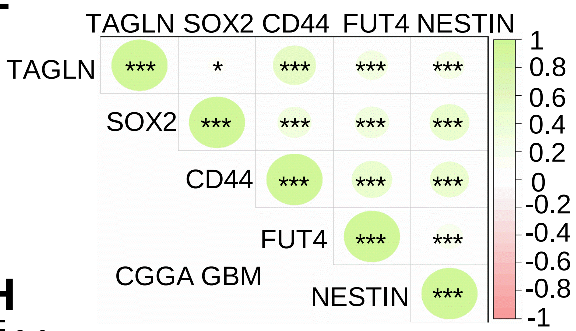
TAGLN SOX2 CD44 FUT4 NESTIN
TAGLN
SOX2
CD44
FUT4
NESTIN
CGGA GBM
***
*
1
0.8
0.6
0.4
0.2
0
-0.2
-0.4
-0.6
-0.8
-1

G

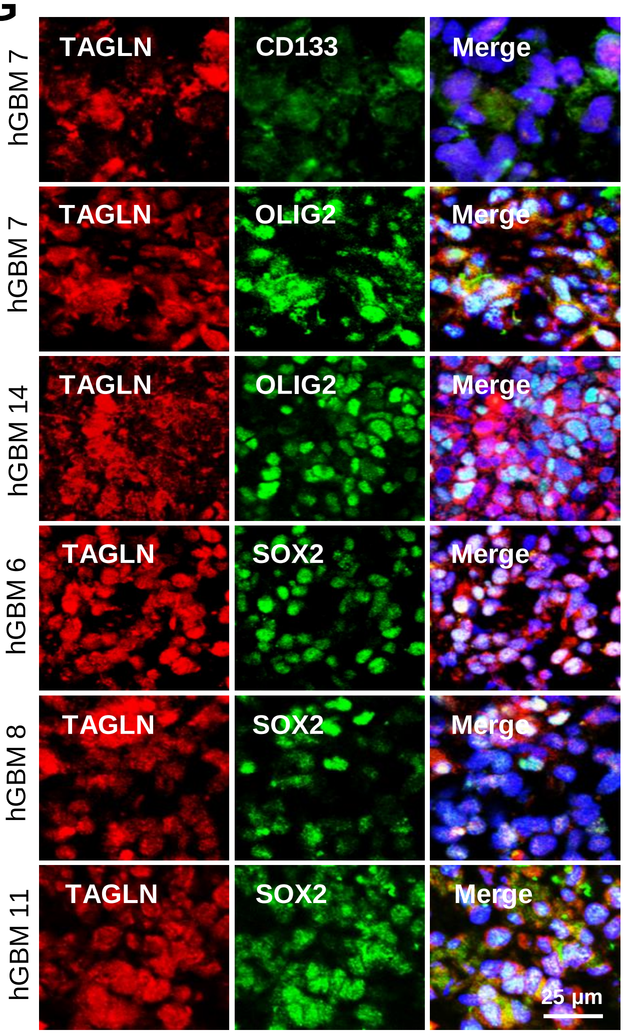
hGBM 7
hGBM 7
hGBM 14
hGBM 6
hGBM 8
hGBM 11
TAGLN
CD133
OLIG2
SOX2
Merge
25 μm

H

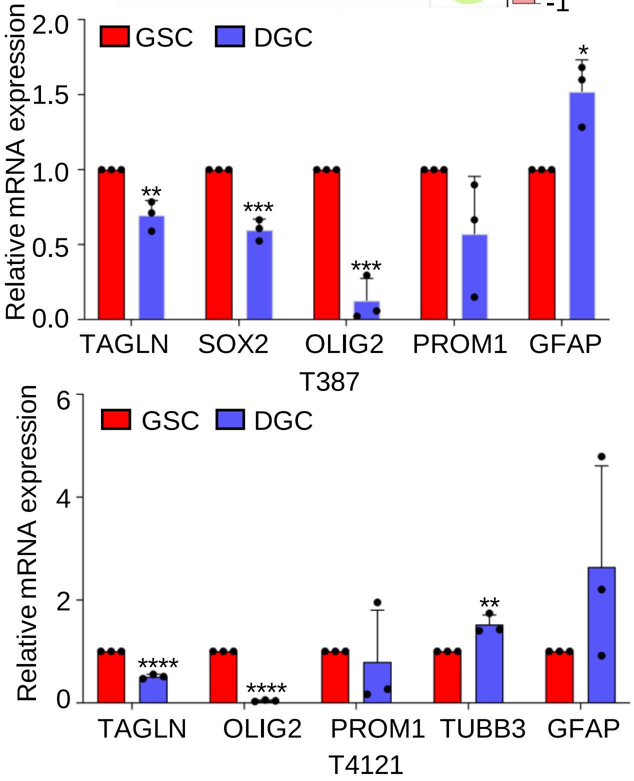
Relative mRNA expression
2.0
1.5
1.0
0.5
0.0
GSC
DGC
**
***
***
*
TAGLN SOX2 OLIG2 PROM1 GFAP
T387
Relative mRNA expression
6
4
2
0
GSC
DGC
****
****
**
TAGLN OLIG2 PROM1 TUBB3 GFAP
T4121

Fig S2. TAGLN is induced high expression in GSCs under hypoxic conditions.

A. Correlation between *TAGLN* and hypoxia-related genes mRNA expression in the TCGA GBM/LGG (IDH-WT) RNA-Seq dataset. The size and color of the dots indicate the degree of correlation. Blank cells show a non-significant correlation.

B. Correlation between *TAGLN* and hypoxia-related genes mRNA expression in the CGGA GBM (IDH-WT) database.

C. HE and IHC staining of human GBM12 sample for CA9, HIF1α, SOX2, and TAGLN in pseudopalisades (upper) and other regions (lower). Scale bar, 100 μm or 50 μm.

D. IF staining of TAGLN with several hypoxia-related proteins, including CA9 and HIF1α in human GBM samples. TAGLN was labeled in red, CA9, or HIF1α in green; and nuclei were counterstained with DAPI (blue). Scale bar, 25 μm.

E. Expression of *TAGLN* and *HIF1A* detected by qRT-PCR in GSCs cultured in 21% O_2 or 1% O_2 for 48 hours.

F. Scatterplot of *TAGLN* expression and GSCs markers expression in CGGA GBM (IDH-WT) database. The size and color of the dots indicate the degree of correlation.

G. IF staining of TAGLN (+) cells with GSCs marked by CD133, OLIG2, and SOX2 in GBM specimens. TAGLN was labeled in red and CD133, SOX2, and OLIG2 were labeled in green. Nuclei were counterstained with DAPI (blue). Scale bar, 25 μm.

H. qRT-PCR for comparison of *TAGLN* expression between GSCs (T387 and T4121) and DGCs. Data are presented as mean ± SD (*$p < 0.05$, **$p < 0.01$, ***$p < 0.001$, ****$p < 0.0001$).

Figure S3

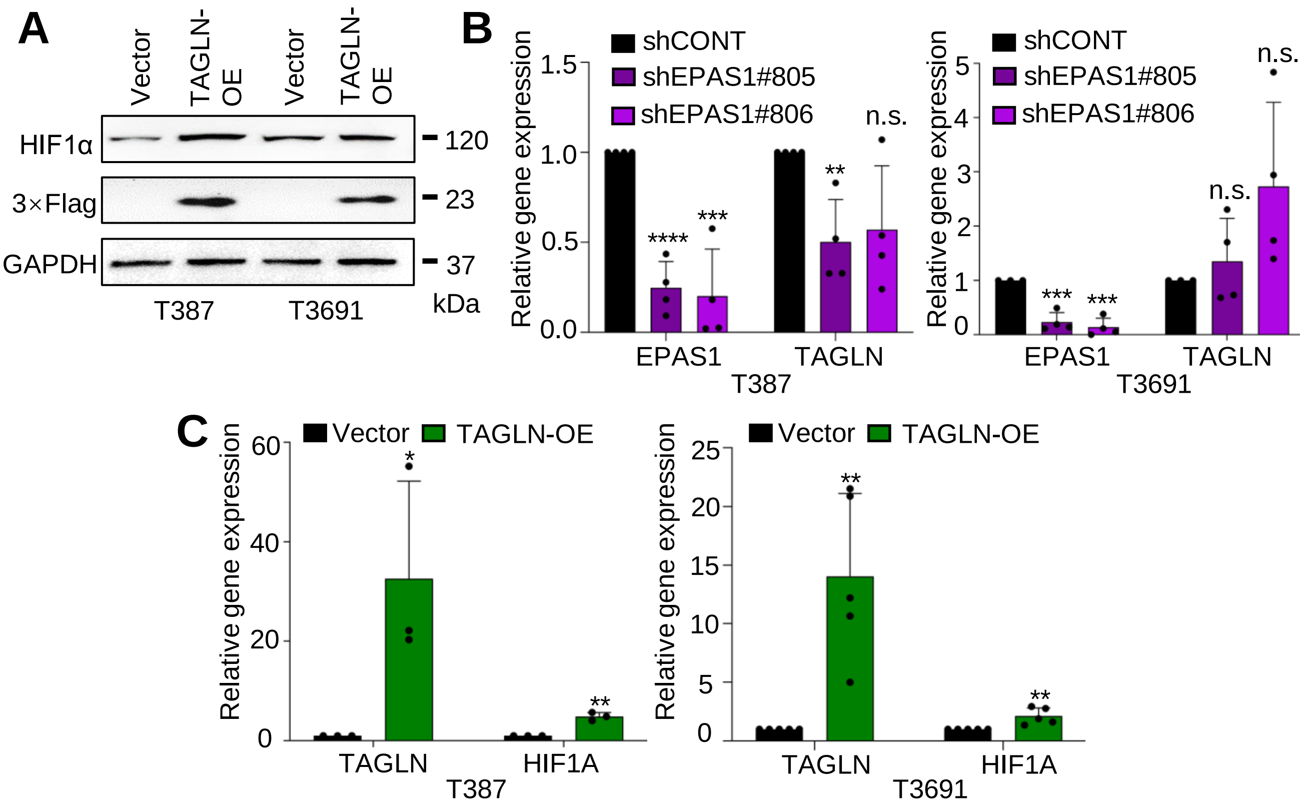

Fig S3. TAGLN binds to HIF1α to form a transcription factor complex under hypoxic conditions.

A. Immunoblot analysis of HIF1α and 3×Flag expression in GSCs (lines T387 and T3691) after transduction with TAGLN-OE or vector control in normoxia.

B. mRNA expression of *EPAS1* and *TAGLN* was assessed by qRT-PCR following EPAS1 knockdown in T387 and T3691 GSCs.

C. qRT-PCR analysis to determine the mRNA expression of *TAGLN* and *HIF1A* in GSCs (T387 and T3691) expressing TAGLN-OE or the vector control. Data are presented as the mean ± SD (*p < 0.05, **p < 0.01,***p < 0.001 ****p < 0.0001, n.s., not significant).

Figure S4

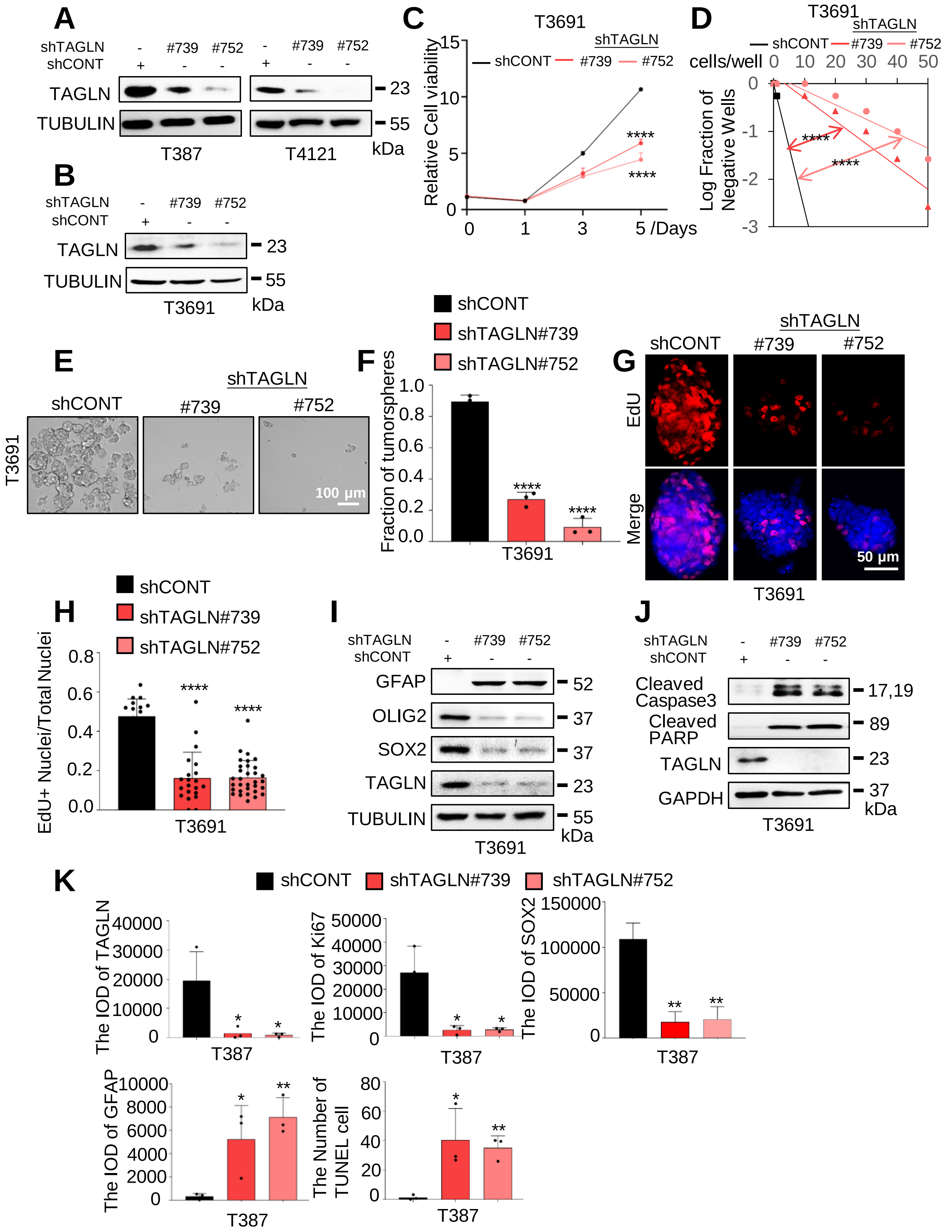

A
shTAGLN - #739 #752 - #739 #752
shCONT + - - + - -
TAGLN
TUBULIN
23
55
kDa
T387
T4121
B
shTAGLN - #739 #752
shCONT + - -
TAGLN
TUBULIN
23
55
T3691
kDa
C
T3691
shTAGLN
shCONT #739 #752
Relative Cell viability
****
****
0 1 3 5 /Days
D
T3691
shTAGLN
shCONT #739 #752
cells/well 0 10 20 30 40 50
Log Fraction of Negative Wells
****
****
E
shCONT
shTAGLN
#739
#752
T3691
100 μm
F
shCONT
shTAGLN#739
shTAGLN#752
Fraction of tumorspheres
****
****
T3691
G
shCONT
shTAGLN
#739
#752
EdU
Merge
50 μm
T3691
H
shCONT
shTAGLN#739
shTAGLN#752
EdU+ Nuclei/Total Nuclei
****
****
T3691
I
shTAGLN - #739 #752
shCONT + - -
GFAP
OLIG2
SOX2
TAGLN
TUBULIN
52
37
37
23
55
kDa
T3691
J
shTAGLN - #739 #752
shCONT + - -
Cleaved Caspase3
Cleaved PARP
TAGLN
GAPDH
17,19
89
23
37
kDa
T3691
K
shCONT shTAGLN#739 shTAGLN#752
The IOD of TAGLN
The IOD of Ki67
The IOD of SOX2
The IOD of GFAP
The Number of TUNEL cell
T387

Fig S4. Ablation of TAGLN impairs the self-renewal and tumorigenicity of GSCs.

A-B. Immunoblot analysis of TAGLN in TAGLN-knockdown T387, T4121, and T3691 GSCs.

C-D. Effects of TAGLN knockdown by shRNAs on cell viability (C) and sphere-forming frequency (D) in T3691 GSCs.

E. Representative bright-field images of neurospheres from T3691 GSCs infected with shTAGLN or shCONT lentivirus. Scale bar, 100 μm.

F. Quantification of the number of neurospheres was shown in Figure (E).

G-H. Representative images of EdU-positive cells (stained red) (G), counted per neurosphere, and quantified (H) after targeting TAGLN. Scale bar, 50 μm.

I. Immunoblot analysis revealed the protein levels of GFAP, OLIG2, and SOX2 in T3691 GSCs transfected with shTAGLN or shCONT.

J. Immunoblot analysis of Cleaved PARP and Cleaved Caspase3 in T3691 GSCs with TAGLN knockdown.

K. Quantification of TAGLN, Ki67, SOX2 and GFAP protein expression levels, and the number of TUNEL cells in nude mouse brain sections after transplantation of T387 GSCs expressing shCONT or shTAGLN. Data are presented as mean ± SD (*$p < 0.05$, **$p < 0.01$, ****$p < 0.0001$).

Figure S5

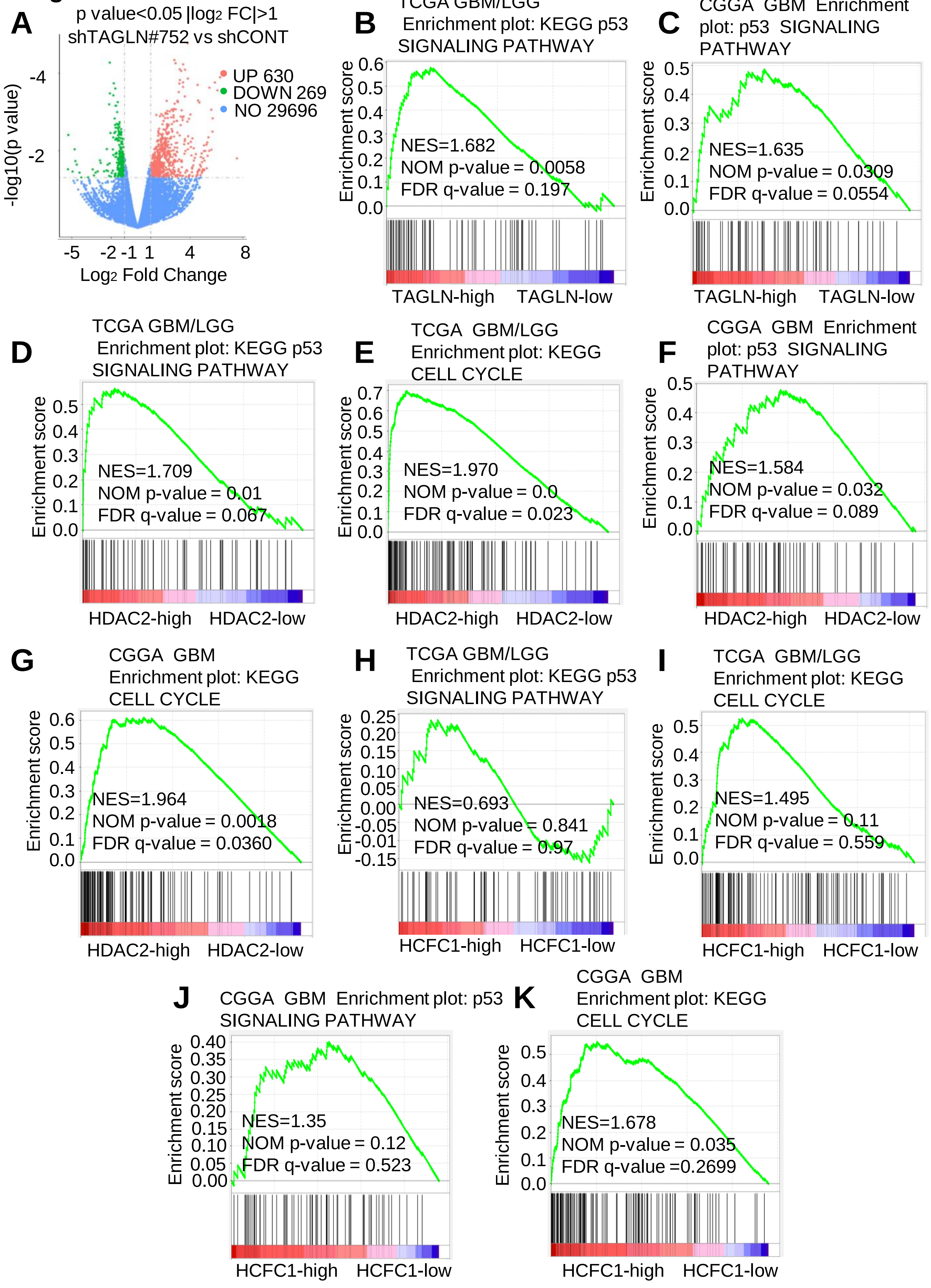

A
p value<0.05 |log2 FC|>1
shTAGLN#752 vs shCONT
-log10(p value)
Log2 Fold Change
UP 630
DOWN 269
NO 29696
B
TCGA GBM/LGG
Enrichment plot: KEGG p53 SIGNALING PATHWAY
Enrichment score
NES=1.682
NOM p-value = 0.0058
FDR q-value = 0.197
TAGLN-high
TAGLN-low
C
CGGA GBM Enrichment plot: p53 SIGNALING PATHWAY
Enrichment score
NES=1.635
NOM p-value = 0.0309
FDR q-value = 0.0554
TAGLN-high
TAGLN-low
D
TCGA GBM/LGG
Enrichment plot: KEGG p53 SIGNALING PATHWAY
Enrichment score
NES=1.709
NOM p-value = 0.01
FDR q-value = 0.067
HDAC2-high
HDAC2-low
E
TCGA GBM/LGG
Enrichment plot: KEGG CELL CYCLE
Enrichment score
NES=1.970
NOM p-value = 0.0
FDR q-value = 0.023
HDAC2-high
HDAC2-low
F
CGGA GBM Enrichment plot: p53 SIGNALING PATHWAY
Enrichment score
NES=1.584
NOM p-value = 0.032
FDR q-value = 0.089
HDAC2-high
HDAC2-low
G
CGGA GBM
Enrichment plot: KEGG CELL CYCLE
Enrichment score
NES=1.964
NOM p-value = 0.0018
FDR q-value = 0.0360
HDAC2-high
HDAC2-low
H
TCGA GBM/LGG
Enrichment plot: KEGG p53 SIGNALING PATHWAY
Enrichment score
NES=0.693
NOM p-value = 0.841
FDR q-value = 0.97
HCFC1-high
HCFC1-low
I
TCGA GBM/LGG
Enrichment plot: KEGG CELL CYCLE
Enrichment score
NES=1.495
NOM p-value = 0.11
FDR q-value = 0.559
HCFC1-high
HCFC1-low
J
CGGA GBM Enrichment plot: p53 SIGNALING PATHWAY
Enrichment score
NES=1.35
NOM p-value = 0.12
FDR q-value = 0.523
HCFC1-high
HCFC1-low
K
CGGA GBM
Enrichment plot: KEGG CELL CYCLE
Enrichment score
NES=1.678
NOM p-value = 0.035
FDR q-value =0.2699
HCFC1-high
HCFC1-low

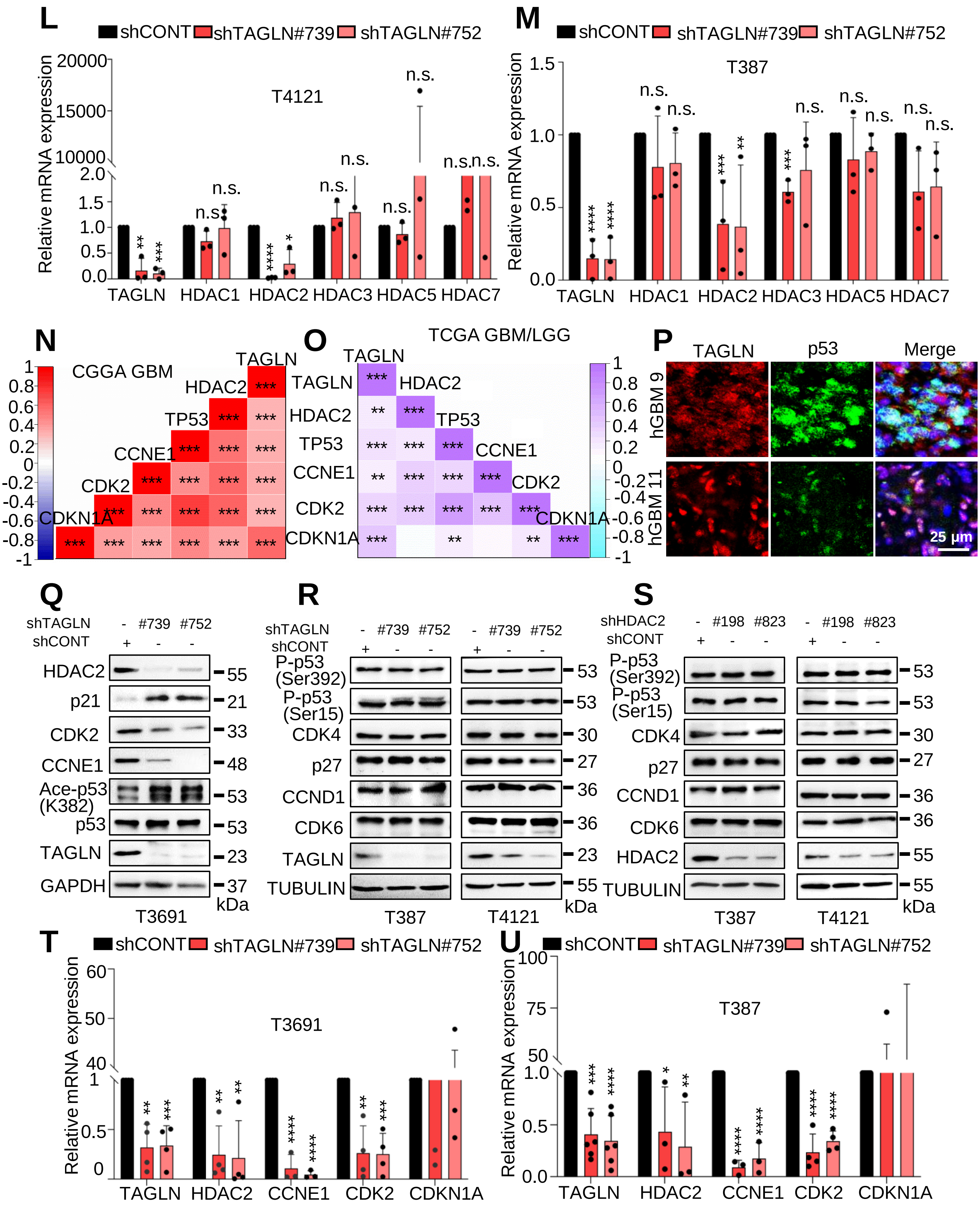
L
shCONT
shTAGLN#739
shTAGLN#752
T4121
Relative mRNA expression
20000
15000
10000
2.0
1.5
1.0
0.5
0.0
TAGLN
HDAC1
HDAC2
HDAC3
HDAC5
HDAC7
n.s.
M
T387
1.5
1.0
0.5
0.0
N
CGGA GBM
TAGLN
HDAC2
TP53
CCNE1
CDK2
CDKN1A
O
TCGA GBM/LGG
P
TAGLN
p53
Merge
hGBM 9
hGBM 11
25 μm
Q
shTAGLN
shCONT
#739
#752
HDAC2
p21
CDK2
CCNE1
Ace-p53 (K382)
p53
TAGLN
GAPDH
55
21
33
48
53
23
37
kDa
T3691
R
P-p53 (Ser392)
P-p53 (Ser15)
CDK4
p27
CCND1
CDK6
TUBULIN
T387
T4121
S
shHDAC2
#198
#823
T
60
50
40
1
0.5
0
TAGLN
HDAC2
CCNE1
CDK2
CDKN1A
U
100
75
50

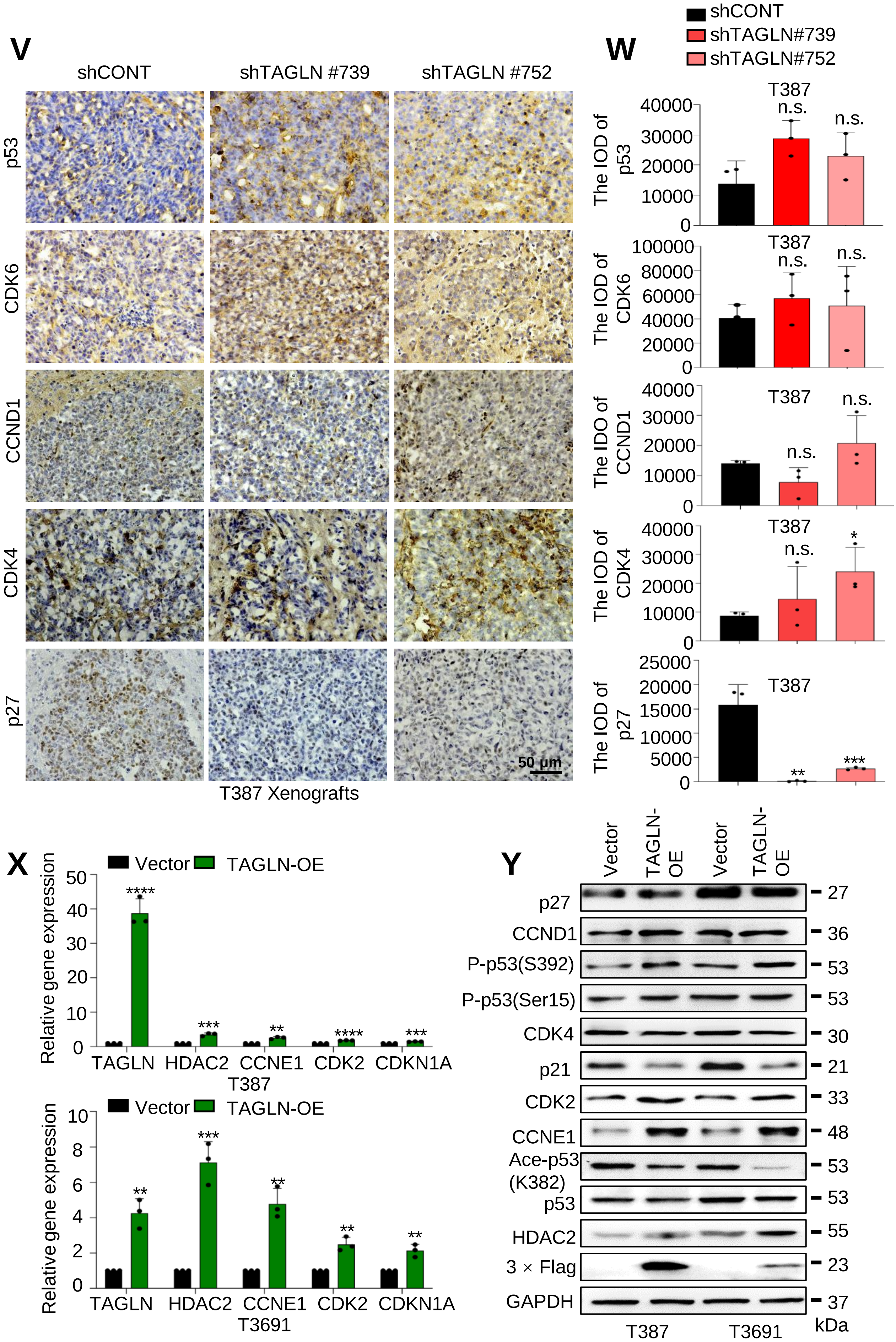

V
shCONT
shTAGLN #739
shTAGLN #752
p53
CDK6
CCND1
CDK4
p27
50 μm
T387 Xenografts
W
shCONT
shTAGLN#739
shTAGLN#752
The IOD of p53
The IOD of CDK6
The IDO of CCND1
The IOD of CDK4
The IOD of p27
T387
n.s.
*
**
***
X
Vector
TAGLN-OE
Relative gene expression
TAGLN
HDAC2
CCNE1
CDK2
CDKN1A
T387
T3691
Y
Vector
TAGLN-OE
p27
CCND1
P-p53(S392)
P-p53(Ser15)
CDK4
p21
CDK2
CCNE1
Ace-p53 (K382)
p53
HDAC2
3 × Flag
GAPDH
27
36
53
53
30
21
33
48
53
53
55
23
37
kDa
T387
T3691

Fig S5. TAGLN-HDAC2 facilitates cell cycle progression by affecting deacetylated p53.

A. Volcano plot of gene expression changes in TAGLN knockdown vs control, obtained from T387 GSCs. After TAGLN knockdown, functional annotation of the upregulated and downregulated genes were shown in red and green at $|\log_2 FC| > 1$ and $p < 0.05$, respectively.

B-K. Significantly enriched p53 signaling pathway and/or cell cycle processes associated with *TAGLN*, *HDAC2 and HCFC1* expression and their nominal p-value, FDR q-value, and normalized enrichment score are shown by gene set enrichment analysis (GSEA).

L-M. qRT-PCR analysis of the indicated genes of the *HDAC* family in T387 and T4121 GSCs after TAGLN knockdown.

N. The expression of *TAGLN* was significantly positively correlated with the expression of *HDAC2*, *TP53*, *CCNE1*, *CDK2*, and *CDKN1A* according to the CGGA GBM (IDH-WT) dataset.

O. The expression of *TAGLN* was significantly positively correlated with the expression of *HDAC2*, *TP53*, *CCNE1*, *CDK2*, and *CDKN1A* according to the TCGA GBM/LGG (IDH-WT) dataset.

P. IF staining indicated the colocalization of TAGLN and p53 in frozen sections of human GBM tissues. TAGLN was labeled in red, p53 in green, and nuclei in blue. Scale bar, 25 μm.

Q. IB analysis of TAGLN, HDAC2, CCNE1, CDK2, p21, ace-p53(K382), and total p53 in T3691 GSCs expressing shCONT and shTAGLN.

R-S. Immunoblot analysis of CCND1, p27, CDK4, CDK6, P-p53(Ser15), and P-p53(Ser392) in T387 and T4121 GSCs with TAGLN or HDAC2 knockdown.

T-U. RNA-seq profile validation by qRT-PCR in T387 and T3691 GSCs.

V. Representative IHC images of p53, CDK6, CCND1, CDK4, and p27 in GBM xenografts derived from T387 GSCs expressing shCONT and shTAGLN. Scale bar, 50 μm.

W. Quantification of the expression levels of p53, CDK6, CCND1, CDK4, and p27 proteins in GBM xenografts in supplementary Fig. S5V.

X. qRT-PCR analysis of *TAGLN*, *HDAC2*, *CCNE1*, *CDK2*, and *CDKN1A* mRNA expression in GSCs expressing TAGLN-OE or the vector.

Y. IB analysis of HDAC2, phosphorylated and acetylated p53, total p53, and several cell cycle proteins in GSCs with or without TAGLN overexpression with a Flag tag (TAGLN-OE). Data are presented as mean ± SD (*p < 0.05, **p < 0.01, ***p < 0.001 **** p < 0.0001, n.s., not significant).

Figure S6

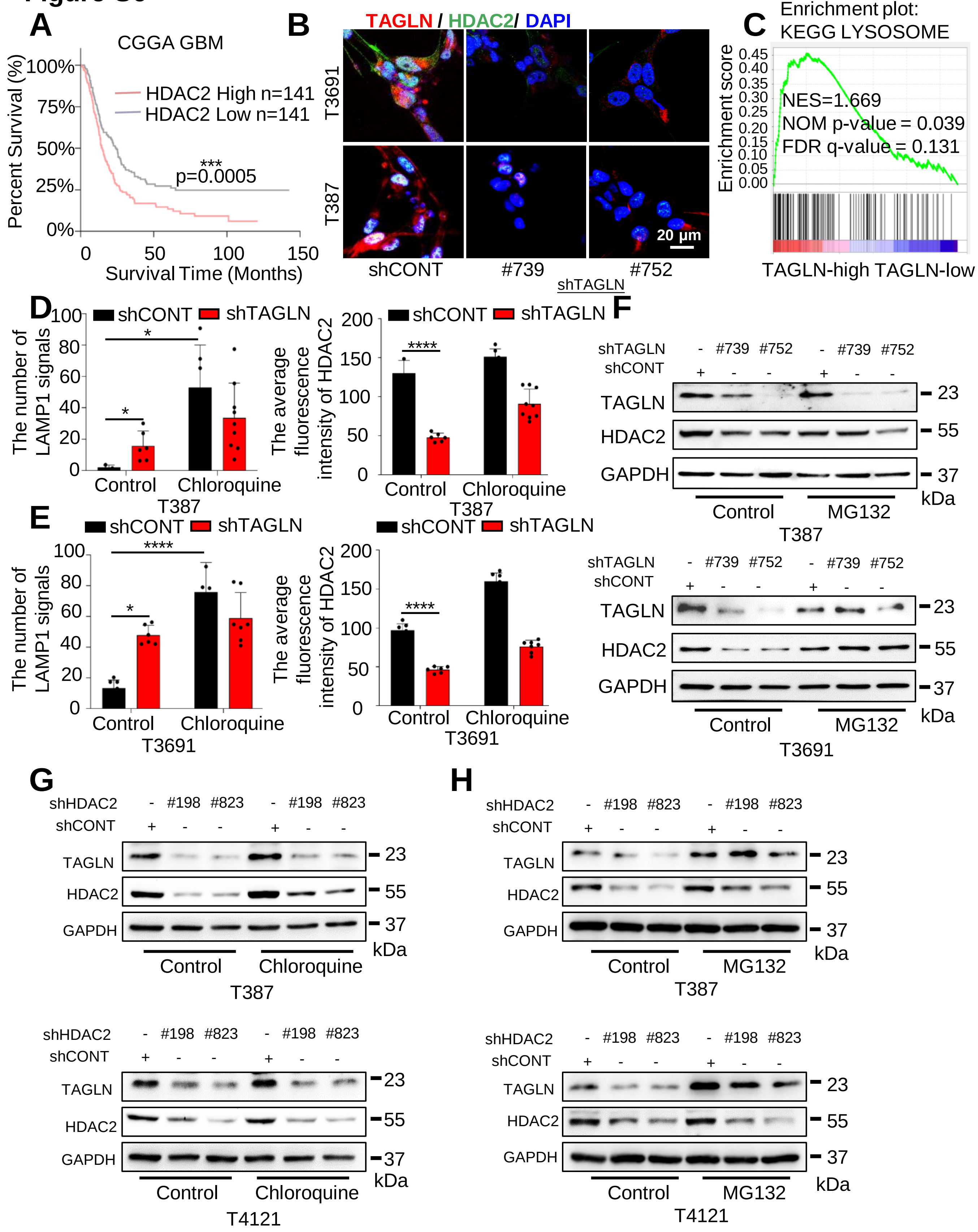

A
CGGA GBM
Percent Survival (%)
HDAC2 High n=141
HDAC2 Low n=141
***
p=0.0005
Survival Time (Months)
B
TAGLN / HDAC2/ DAPI
T3691
T387
20 µm
shCONT
#739
#752
shTAGLN
C
Enrichment plot:
KEGG LYSOSOME
Enrichment score
NES=1.669
NOM p-value = 0.039
FDR q-value = 0.131
TAGLN-high TAGLN-low
D
shCONT shTAGLN
The number of LAMP1 signals
Control Chloroquine
T387
The average fluorescence intensity of HDAC2
E
T3691
F
shTAGLN
shCONT
TAGLN
HDAC2
GAPDH
kDa
Control MG132
G
shHDAC2
shCONT
#198 #823
T4121
H

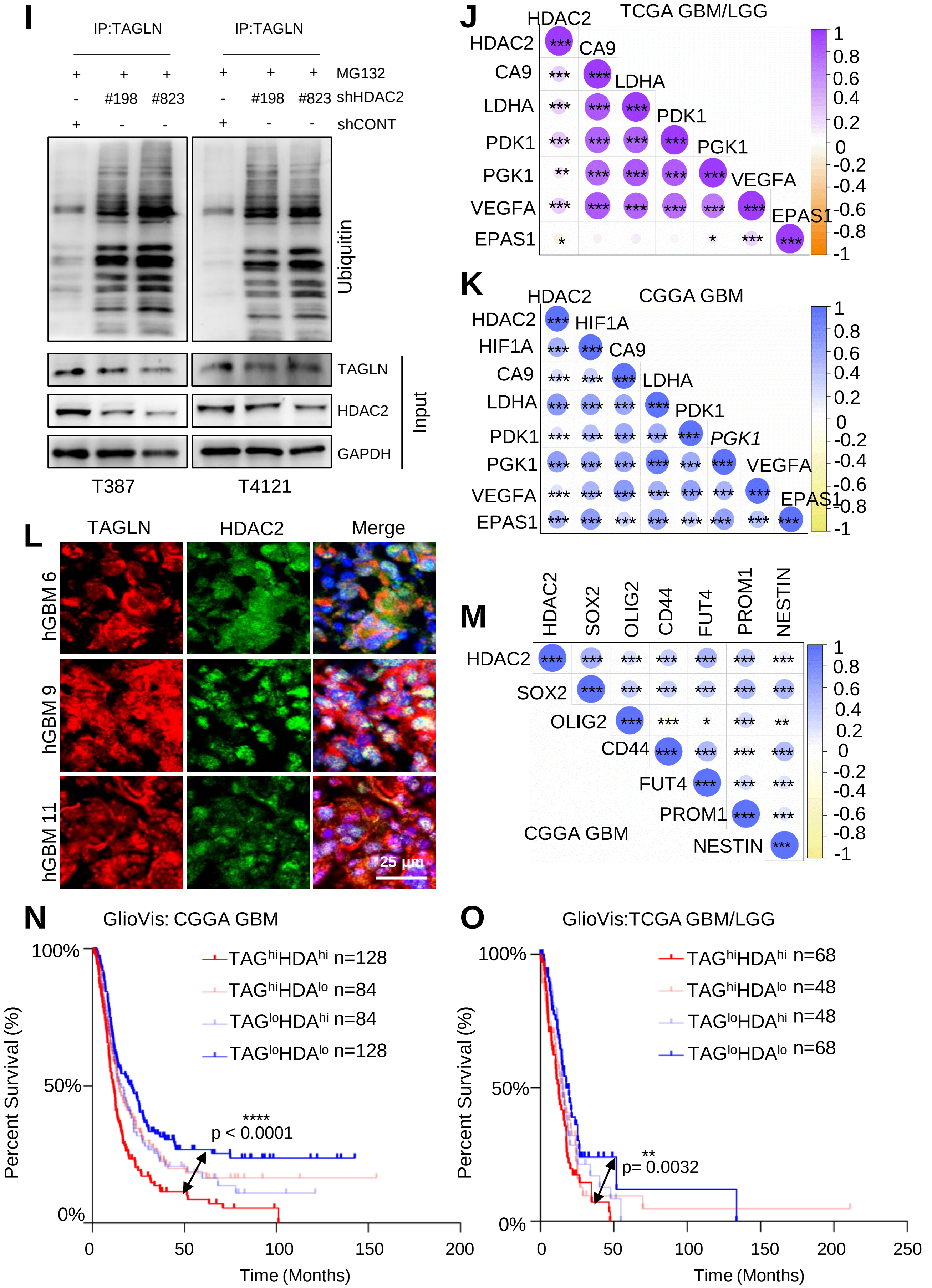
I
IP:TAGLN
IP:TAGLN
MG132
shHDAC2
shCONT
#198
#823
Ubiquitin
TAGLN
HDAC2
GAPDH
Input
T387
T4121
J
TCGA GBM/LGG
HDAC2
CA9
LDHA
PDK1
PGK1
VEGFA
EPAS1
K
CGGA GBM
HIF1A
L
TAGLN
HDAC2
Merge
hGBM 6
hGBM 9
hGBM 11
25 μm
M
SOX2
OLIG2
CD44
FUT4
PROM1
NESTIN
CGGA GBM
N
GlioVis: CGGA GBM
TAGhiHDAhi n=128
TAGhiHDAlo n=84
TAGloHDAhi n=84
TAGloHDAlo n=128
p < 0.0001
Percent Survival (%)
Time (Months)
O
GlioVis:TCGA GBM/LGG
TAGhiHDAhi n=68
TAGhiHDAlo n=48
TAGloHDAhi n=48
TAGloHDAlo n=68
p= 0.0032
Percent Survival (%)
Time (Months)

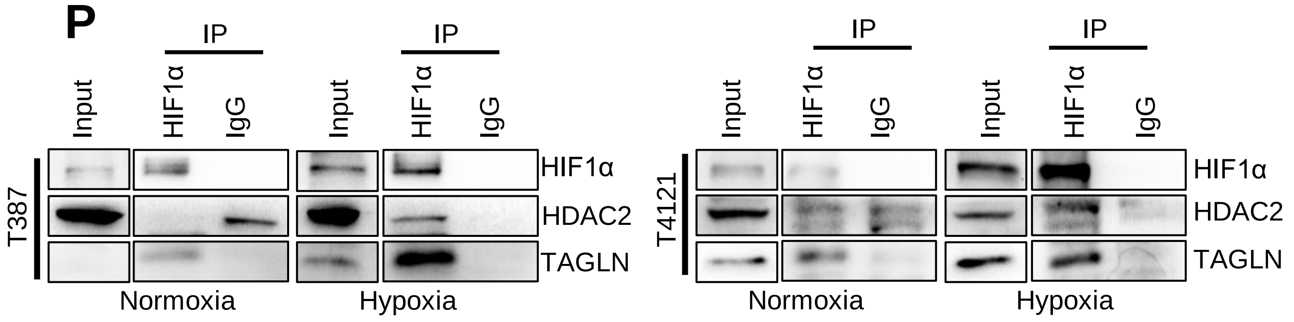

Fig S6. TAGLN binds and regulates HDAC2 under hypoxic conditions.

A. Kaplan–Meier curve showing patient survival based on *HDAC2* mRNA expression in the CGGA GBM (IDH-WT) dataset.

B. IF staining showed decreased expression of TAGLN and HDAC2 in GSCs with TAGLN knockdown. TAGLN was labeled in red, HDAC2 was labeled in green, and nuclei were counterstained with DAPI (blue). Scale bar, 20 μm.

C. GSEA of high *TAGLN* expression compared to low *TAGLN* expression. The gene sets showed the presence of lysosome-associated genes. GSEA of hallmark gene sets used a ranked gene list weighted by gene expression within TCGA GBM/LGG (IDH-WT) database.

D-E. The total number of green LAMP1 dots in each cell under each condition was quantified (left) and quantification of the mean fluorescence intensity of HDAC2 per cell in different treatment groups (right).

F. IB analysis of TAGLN and HDAC2 in GSCs transduced with shCONT or shTAGLN and treated with the proteasome inhibitor MG132 for 2 h under hypoxia.

G-H. IB showing degradation pathways of the TAGLN protein in GSCs with or without HDAC2 knockdown.

I. Co-IP of TAGLN and then western blotted with anti-ubiquitin in T387 and T4121 GSCs.

J-K. Positive correlation between *HDAC2* and hypoxia-related genes in the TCGA GBM/LGG (IDH-WT) and CGGA (IDH-WT) GBM datasets. The size and color of the dots indicate the degree of correlation.

L. Representative IF images showing the colocalization of TAGLN and HDAC2 in GBM specimens. Scale bar, 25 μm.

M. Positive correlation between *HDAC2* mRNA expression and *SOX2, OLIG2, CD44, FUT4, PROM1,* and *NESTIN* levels in CGGA GBM (IDH-WT) dataset. Size and color indicate the degree of correlation.

N-O. The survival cruves of $TAG^{hi}HDA^{hi}$, $TAG^{hi}HDA^{lo}$, $TAG^{lo}HDA^{hi}$ and TAG^{lo} HDA^{lo} groups from CGGA GBM (IDH-WT) and TCGA GBM/LGG (IDH-WT) were compared.

P. Co-IP of TAGLN, HDAC2, and HIF1α with the HIF1α-specific antibody from GSCs cell lysates under normoxia and hypoxia.

(*p < 0.05, **p < 0.01, ***p < 0.001, ****p < 0.0001).

Figure S7

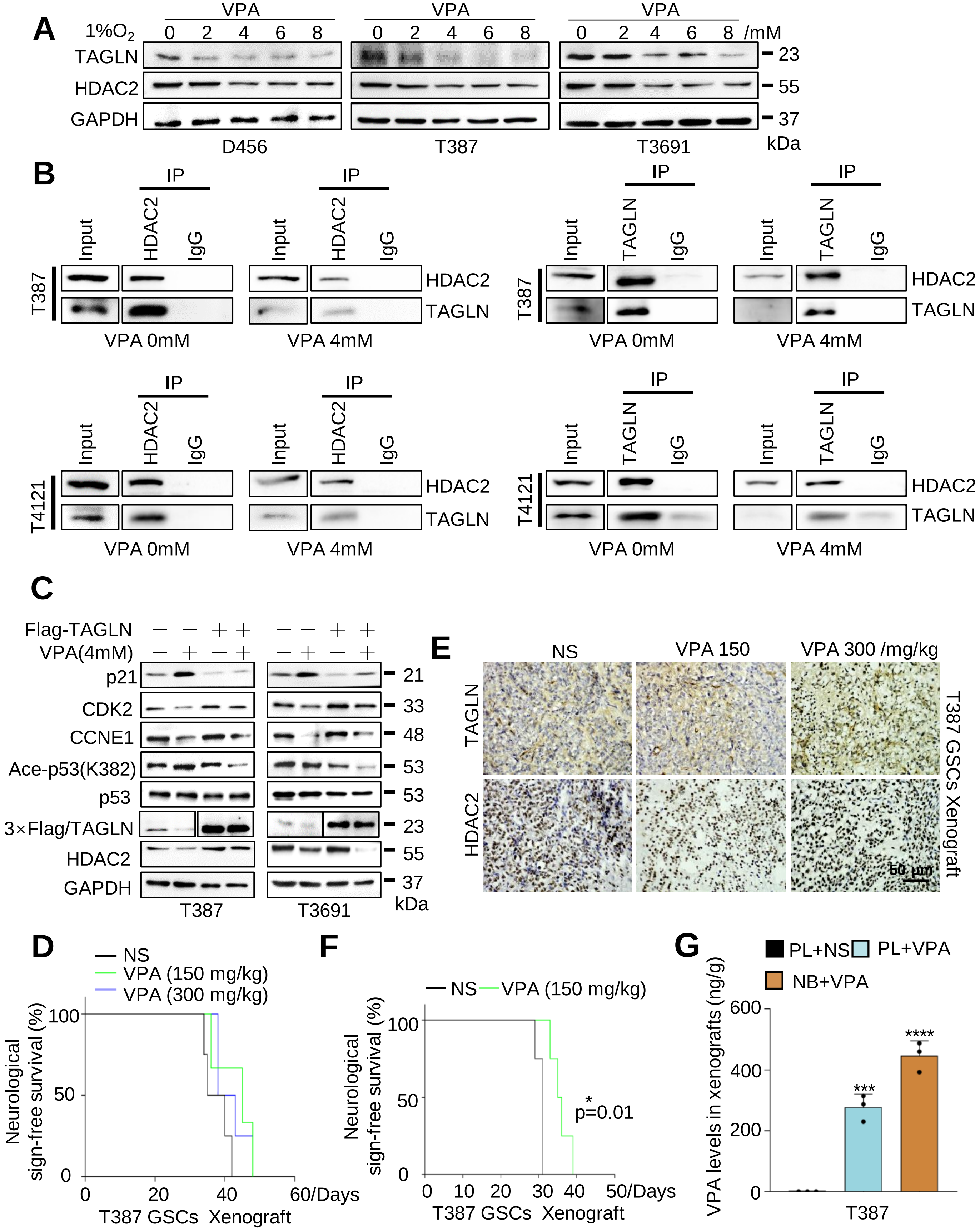

A
VPA
1%O2 0 2 4 6 8 /mM
TAGLN
HDAC2
GAPDH
23
55
37
kDa
D456
T387
T3691
B
IP
Input
HDAC2
IgG
TAGLN
T387
T4121
VPA 0mM
VPA 4mM
C
Flag-TAGLN
VPA(4mM)
p21
CDK2
CCNE1
Ace-p53(K382)
p53
3×Flag/TAGLN
HDAC2
GAPDH
21
33
48
53
53
23
55
37
kDa
T387
T3691
E
NS
VPA 150
VPA 300 /mg/kg
TAGLN
HDAC2
T387 GSCs Xenograft
50 μm
D
NS
VPA (150 mg/kg)
VPA (300 mg/kg)
Neurological sign-free survival (%)
100
50
0
0 20 40 60/Days
T387 GSCs Xenograft
F
NS
VPA (150 mg/kg)
Neurological sign-free survival (%)
100
50
0
0 10 20 30 40 50/Days
*
p=0.01
T387 GSCs Xenograft
G
PL+NS
PL+VPA
NB+VPA
VPA levels in xenografts (ng/g)
600
400
200
0
***
****
T387

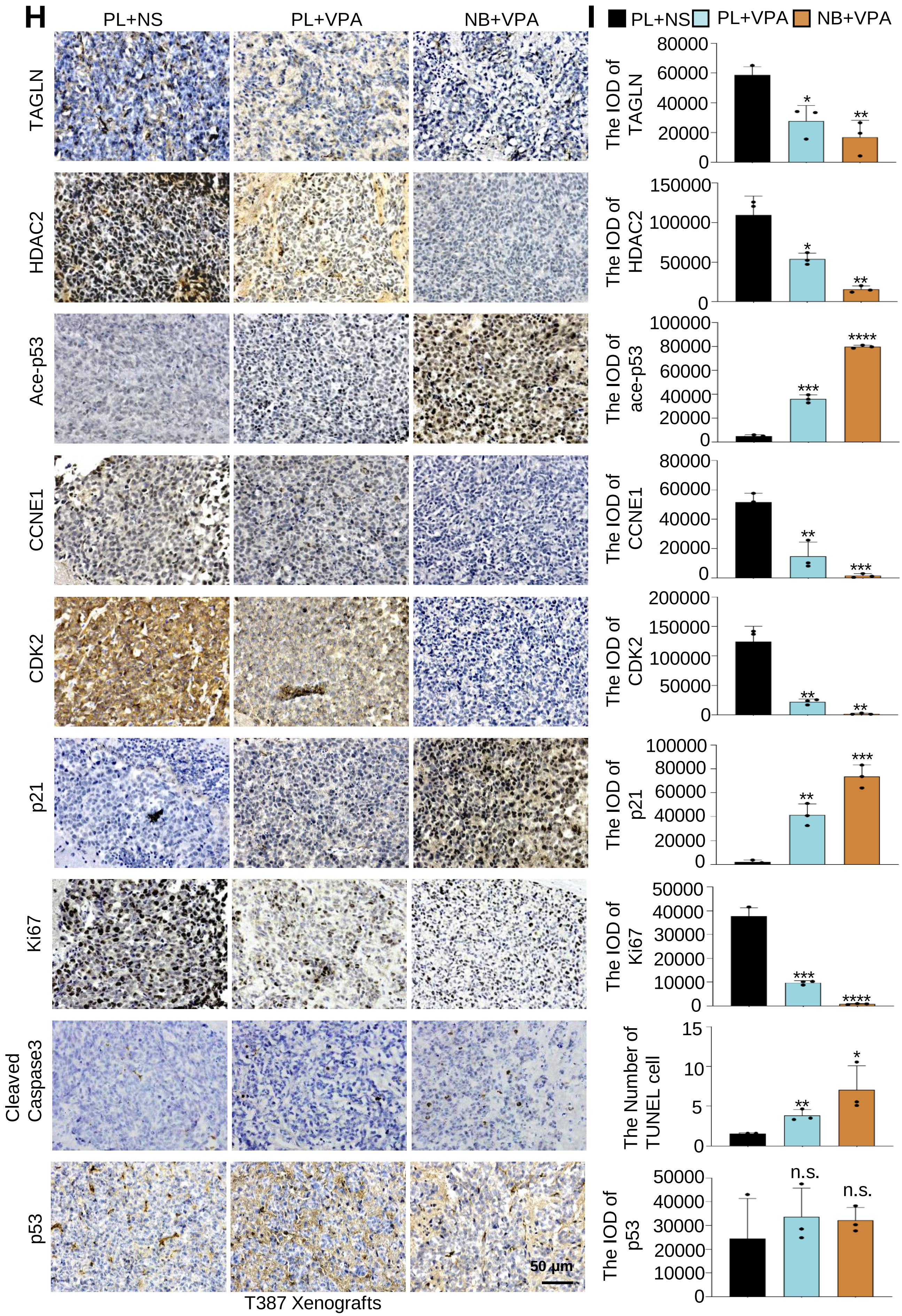
H
PL+NS
PL+VPA
NB+VPA
TAGLN
HDAC2
Ace-p53
CCNE1
CDK2
p21
Ki67
Cleaved Caspase3
p53
50 μm
T387 Xenografts
I
PL+NS
PL+VPA
NB+VPA
The IOD of TAGLN
The IOD of HDAC2
The IOD of ace-p53
The IOD of CCNE1
The IOD of CDK2
The IOD of p21
The IOD of Ki67
The Number of TUNEL cell
The IOD of p53
n.s.

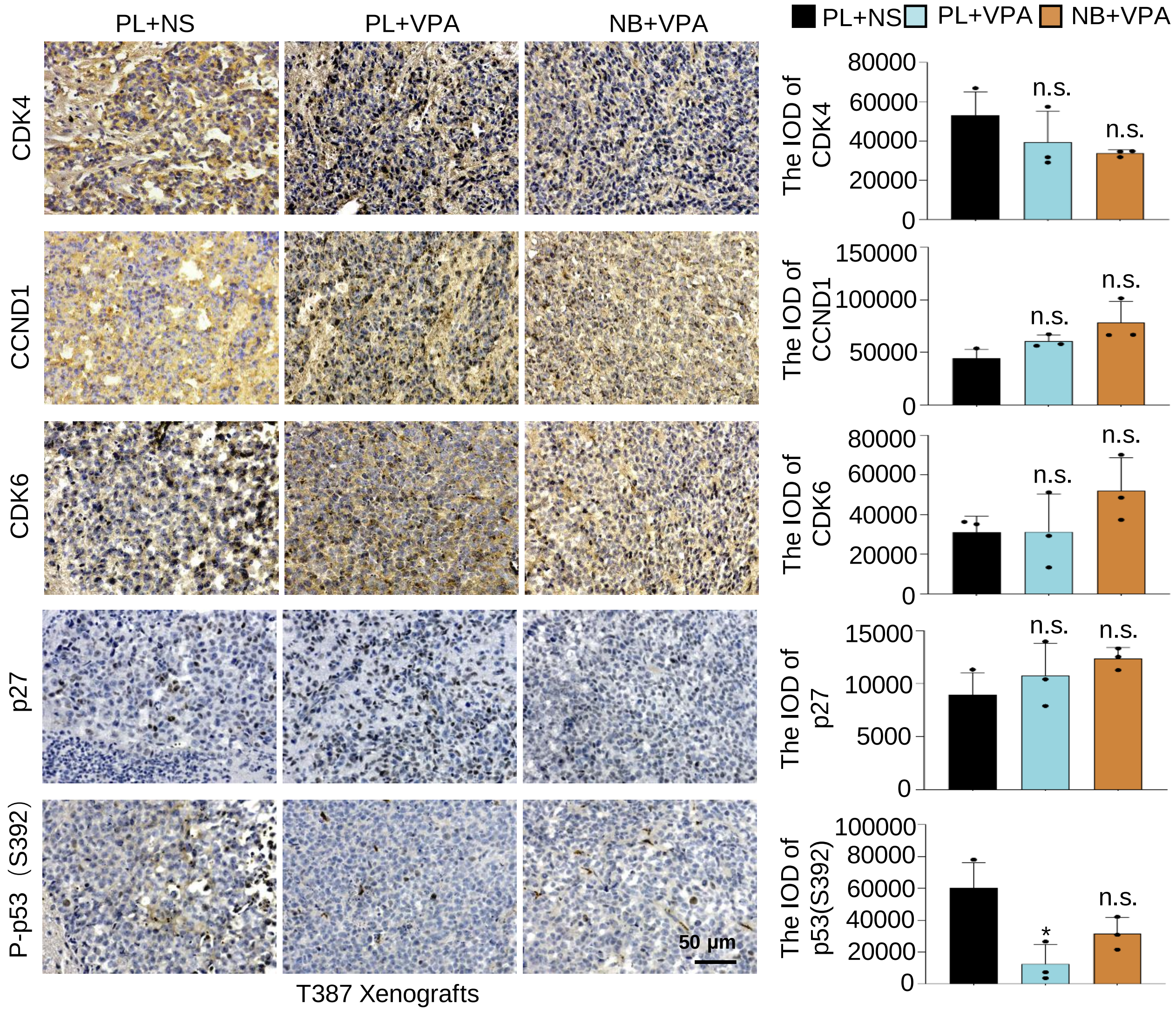

PL+NS
PL+VPA
NB+VPA
CDK4
CCND1
CDK6
p27
P-p53（S392）
50 μm
T387 Xenografts
PL+NS
PL+VPA
NB+VPA
The IOD of CDK4
The IOD of CCND1
The IOD of CDK6
The IOD of p27
The IOD of p53(S392)
n.s.
*

Fig S7. Targeting TAGLN/HDAC2 attenuates the tumor-initiating ability of GSCs.

A. GSC lines (D456, T387, and T3691) were treated with different doses of VPA, and TAGLN and HDAC2 protein levels were analyzed by immunoblotting.

B. Co-IP analysis of TAGLN and HDAC2 in T387 and T4121 GSCs with or without 4mM VPA incubation.

C. Immunoblot analysis of 3×Flag/TAGLN, HDAC2, acetylated p53 at K382, total p53, CCNE1, CDK2, p21, and GAPDH in T387 or T3691 GSCs with and without TAGLN overexpression treated with NS or VPA.

D. Kaplan-Meier curves of nude mice bearing T387 GSCs orthotopic xenografts with the indicated treatments.

E. Representative IHC images of TAGLN and HDAC2 in GBM xenografts derived from T387 GSCs treated with VPA at different concentrations. Scale bar, 50 μm.

F. Kaplan-Meier curves were drawn to assess the burden of tumor progression after intraperitoneal injection of NS or VPA (150 mg/kg) in tumor-bearing mice for 2 weeks.

G. LC-MS-based quantification of VPA in xenografts of tumor-bearing mice.

H-I. Representative IHC images of indicated proteins in the xenografts after the indicated treatments and quantified. Scale bar, 50 μm. Data are presented as mean ± SD.

(* $p < 0.05$, ** $p < 0.01$, *** $p < 0.001$, **** $p < 0.0001$).

Table S1. Related to methods. GBM patient information.

Serial number	Age (years old)	gender
hSAH1	54	female
hGBM2	48	male
hGBM3	45	male
hGBM4	30	male
hGBM5	53	female
hGBM6	49	male
hGBM7	52	female
hGBM8	25	male
hGBM9	11	female
hGBM10	51	male
hGBM11	54	male
hGBM12	57	female
hGBM13	51	male
hGBM14	37	male
hGBM15	51	male
hGBM16	32	male
hGBM17	10	male
hGBM18	55	male
hGBM19	28	male
hGBM20	52	female
hGBM21	51	male

Table S2. Related to methods. shRNA sequences.

Serial number	shRNA	Sequence
TRCN0000003808	shHIF1α 808-F	CCGG-CCGCTGGAGACACAATCATAT-CTCGAG-ATATGATTGTGTCTCCAGCGG-TTTTT
	shHIF1α 808-R	AATTCAAAAA-CCGCTGGAGACACAATCATAT-CTCGAG-ATATGATTGTGTCTCCAGCGG
TRCN0000003809	shHIF1α 809-F	CCGG-CCAGTTATGATTGTGAAGTTA-CTCGAG-TAACTTCACAATCATAACTGG-TTTTT
	shHIF1α 809-R	AATTCAAAAA-CCAGTTATGATTGTGAAGTTA-CTCGAG-TAACTTCACAATCATAACTGG
TRCN0000003805	shHIF2α 805-F	CCGG-GCGCAAATGTACCCAATGATA-CTCGAG-TATCATTGGGTACATTTGCGC-TTTTT
	shHIF2α 805-R	AATTCAAAAA-GCGCAAATGTACCCAATGATA-CTCGAG-TATCATTGGGTACATTTGCGC
TRCN0000003806	shHIF2α 806-F	CCGG-CAGTACCCAGACGGATTTCAA-CTCGAG-TTGAAATCCGTCTGGGTACTG-TTTTT
	shHIF2α 806-R	AATTCAAAAA-CAGTACCCAGACGGATTTCAA-CTCGAG-TTGAAATCCGTCTGGGTACTG
TRCN0000307739	shTAGLN 739-F	CCGG-GCATGTCATTGGCCTTCAGAT-CTCGAG-ATCTGAAGGCCAATGACATGC-TTTTTG
	shTAGLN 739-R	AATTCAAAAA-GCATGTCATTGGCCTTCAGAT-CTCGAG-ATCTGAAGGCCAATGACATGC
TRCN0000072752	shTAGLN 752-F	CCGG-GAGTGGATCATAGTGCAGTGT-CTCGAG-ACACTGCACTATGATCCACTC-TTTTTG
	shTAGLN 752-R	AATTCAAAAA-GAGTGGATCATAGTGCAGTGT-CTCGAG-ACACTGCACTATGATCCACTC
TRCN0000195198	shHDAC2 198-F	CCGG-CAGACTGATATGGCTGTTAAT-CTCGAG-ATTAACAGCCATATCAGTCTG-TTTTTG
	shHDAC2 198-R	AATTCAAAAA-CAGACTGATATGGCTGTTAAT-CTCGAG-ATTAACAGCCATATCAGTCTG
TRCN0000004823	shHDAC2 823-F	CCGG-GCAAATACTATGCTGTCAATT-CTCGAG-AATTGACAGCATAGTATTTGC-TTTTTG
	shHDAC2 823-R	AATTCAAAAA-GCAAATACTATGCTGTCAATT-CTCGAG-AATTGACAGCATAGTATTTGC

Table S3. Related to methods. Human qRT-PCR and ChIP primer sequences used in this study.

Genes	Forward primer (5' - 3')	Reverse primer (5' - 3')
TAGLN	CCGTGGAGATCCCAACTGG	CCATCTGAAGGCCAATGACAT
HIF1A	AGTCTAGAGATGCAGCAAGATCTC	TTCCTCATGGTCACATGGATGAGT
EPAS1	CGACAATGACAGCTGACAAGGAG	TTGGTGACCGTGCACTTCATCCTC
SOX2	GCCGAGTGGAAACTTTTGTCG	GGCAGCGTGTACTTATCCTTCT
OLIG2	CAAGAAGCAAATGACAGAGCCGGA	TGGTGAGCATGAGGATGTAGTTGC
PROM1	CTTACGGCACTCTTCACCTG	TCCCTGTGCGTTGAAGTATC
TUBB3	ACTACAACGAGGCCTCTTCTCAC	TTGTTGCCGGCCCCACTCTGACC
GFAP	CTGGAGAGGAAGATTGAGTCGC	ACGTCAAGCTCCACATGGACCT
GAPDH	GTCTCCTCTGACTTCAACAGCG	ACCACCCTGTTGCTGTAGCCAA
ACTB	AGAAAATCTGGCACCACACC	AGAGGCGTACAGGGATAGCA
HDAC1	GGTCCAAATGCAGGCGATTCCT	TCGGAGAACTCTTCCTCACAGG
HDAC2	ATGGCGTACAGTCAAGGAGG	TGCGGATTCTATGAGGCTTCA
HDAC3	AGATGCGCCTGTGTAACGC	GCAAGGCTTCACCAAGAGTCT
HDAC5	CGCTGAGAATGGCTTTACTGGC	GTGTAGAGGCTGAACTGGTTGG
HDAC7	TCCTGGCACAGCGGATGTTTGT	TGAAGGCGAGGTCAGTGACACT
CCNE1	TGTGTCCTGGATGTTGACTGCC	CTCTATGTCGCACCACTGATACC
CDK2	CCAGGAGTTACTTCTATGCCTGA	TTCATCCAGGGGAGGTACAAC
CDKN1A	AGGTGGACCTGGAGACTCTCAG	TCCTCTTGGAGAAGATCAGCCG
ChIP primers, *HIF1A* binding site1	GGCAGGGTCCTGTCCATAAAA	CATGCTTTGGAGATGGAGCG
ChIP primers, *HIF1A* binding site2	CTCCCTGATGCGGCGTTTAT	AACTCCCTCCTCTTGGAGCC
ChIP primers, *HIF1A* binding site3	GCTTCAGGAAGGGATGTGCT	ACTTGCCTGAAGACTGGACG
ChIP primers, *TAGLN* binding site1	TGTGGCCATTGTAAAAACTCATGT	GGGTAGGAGATGGAGATGCAATC
